# Valorizing Pearl Mussel (*Hyriopsis cumingii*) into Hydrolysates: Impact of Different Ultrasonic Pretreatments and Proteases on Its Physicochemical and Functional Properties

**DOI:** 10.3390/md24100348

**Published:** 2026-10-02

**Authors:** Shangkun Li, Yurui Zhang, Yan Li, Ji Shi, Qianqian Liang, Xin Jiang, Zhiyi Bai, Wenzheng Shi

**Affiliations:** 1 College of Food Science and Technology, Shanghai Ocean University, Shanghai 201306, China; 18736192663@163.com (S.L.); 13162885465@163.com (Y.Z.); lyangdou@163.com (Y.L.); 13523474726@163.com (J.S.); lqq18152566062@163.com (Q.L.); 13053523375@163.com (X.J.); 2 Shanghai Collaborative Innovation Center for Cultivating Elite Breeds and Green-Culture of Aquaculture Animals, Shanghai 201306, China; zybai@shou.edu.cn; 3 National R&D Branch Center for Freshwater Aquatic Products Processing Technology (Shanghai), Shanghai 201306, China

**Keywords:** pearl mussel, ultrasound-assisted, hydrolysate, functional properties

## Abstract

Pearl mussel (*Hyriopsis cumingii*) meat is an underutilized byproduct. In this study, the effects of ultrasonic pretreatment (0–600 W) and protease type (flavorzyme, alcalase, bromelain, and trypsin) on the physicochemical, bioactivity, and functional properties of pearl mussel hydrolysates were systematically investigated. The results demonstrated that ultrasonic pretreatment increased the degree of hydrolysis (DH), with alcalase hydrolysates exhibiting the highest DH value (37.94%). Glutamic acid, aspartic acid, and leucine were identified as the predominant components. Specifically, at 300 W, the alcalase hydrolysate (UAH-3) exhibited excellent antioxidant activity. In contrast, at 600 W, the flavorzyme hydrolysate (UFH-6) and bromelain hydrolysate (UBH-6) demonstrated the highest α-glucosidase inhibitory and emulsion activity. Overall, these findings indicate that the combination of ultrasonication and enzymatic hydrolysis is a promising strategy for enhancing the utilization of pearl mussel byproducts and provides a solid theoretical basis for their application in functional foods and nutraceuticals.

## 1. Introduction

The pearl mussel (*Hyriopsis cumingii*) is a freshwater pearl mussel unique to China, primarily used for pearl cultivation and widely farmed across the country [1]. Notably, pearl harvesting produces substantial byproducts, including meat, mantle, and secretions, which are often underutilized or discarded [2]. According to the United Nations “World Population Prospects 2024”, the global population is projected to increase from approximately 8.2 billion in 2024 to around 10.3 billion by the mid-2080s, highlighting the necessity of paying greater attention to food byproducts [3]. Mussel meat (accounting for 35%) is the primary byproduct of the pearl mussel. It is rich in protein and exhibits an amino acid profile superior to that of plant proteins, demonstrating considerable nutritional potential [4]. However, its relatively tough texture and distinct earthy odor limit its application in the food industry. Consequently, enhancing the utilization of pearl mussel meat has become an urgent challenge that requires immediate attention.

Enzymatic hydrolysis is considered a safe, controllable, and efficient approach for processing byproducts. By cleaving peptide bonds within proteins, enzymatic hydrolysis modifies protein structure, molecular weight, and amino acid composition, thereby influencing the functional properties of the resulting products [5]. Previous studies have reported that bovine liver hydrolysates prepared via enzymatic hydrolysis exhibit notable antioxidant activity [6]. Another study demonstrated that, compared with unhydrolyzed porcine liver, porcine liver hydrolysates showed significantly enhanced emulsifying and antibacterial activities against Staphylococcus aureus and Candida albicans [7]. In recent years, shellfish hydrolysates have emerged as a research hotspot due to their excellent bioactivity and potential as functional food ingredients. For example, scallop protein hydrolysates contain a high proportion of antioxidant amino acids (35.25%) and can effectively inhibit H_2_O_2_-induced ROS production [8]. Antioxidant peptides purified from hydrolysates of the pearl shell (*Pinctada martensii*) significantly increased HepG2 cell viability and exhibited strong binding affinity with proteins involved in antioxidant pathways [9]. To our knowledge, no studies have reported on the bioactivity and functionality of hydrolysates derived from the pearl mussel meat. Furthermore, the type of protease is a critical factor influencing the physicochemical properties and functionality of hydrolysates [10]. Proteases derived from animals, plants, and microorganisms exhibit distinct cleavage properties. For instance, flavorzyme preferentially cleaves peptide bonds between leucine and proline, or proline and proline [6]; alcalase, as a serine endopeptidase, possesses exceptional hydrolytic capacity [11]; bromelain, a cysteine protease, exhibits endopeptidase activity [12]; and trypsin primarily acts on the C-terminal peptide bonds of lysine and arginine residues [13]. Wang et al. [5] analyzed hempseed protein hydrolysates prepared using seven different proteases and found that papain hydrolysates with higher DH exhibited excellent surface hydrophobicity but poor emulsion activity. In contrast, trypsin hydrolysates with moderate DH demonstrated good emulsion activity and emulsion stability. Therefore, elucidating the relationship between protease type, and the structure and functionality of pearl mussel hydrolysates is crucial, as it will guide the selection of appropriate proteases for targeted applications.

However, the limitations of traditional enzymatic hydrolysis have become increasingly apparent, primarily due to prolonged hydrolysis times, low enzyme efficiency, and limited biological activity of the products. In recent years, researchers have explored new technologies, including ultrasound [14], microwaves [15], pulsed electric fields [16], and cold plasma [17], for pretreating raw materials or assisting enzymatic hydrolysis, with most studies reporting positive outcomes. Among these, the combination of ultrasound and enzymatic hydrolysis is considered particularly promising due to its simplicity, environmental friendliness, and high efficiency [18]. The shear forces, pressure, and temperature fluctuations induced by ultrasound can alter substrate structures, creating additional sites for enzymatic hydrolysis and thereby enhancing both the functionality and biological activity of the hydrolysates [19]. A related study found that zein hydrolysates prepared via combined ultrasound and alcalase treatment exhibited significantly higher α-glucosidase inhibitory activity than those treated with enzymatic hydrolysis alone [20]. Similarly, ultrasonic-assisted enzymatic hydrolysis markedly improved the capacity of hydrolysates from three plant proteins (broad bean, lentil bean, and mung bean isolated proteins) to protect against oxidative DNA damage and to exhibit ACE inhibitory activity [21]. Yu et al. [22] investigated the effects of different ultrasonic powers (0 W, 300 W, 500 W, and 800 W) on Acheta domesticus protein hydrolysates. Moderate ultrasonic treatment (300 W and 500 W) significantly enhanced the antioxidant activity, whereas excessively high power (800 W) reduced cavitation efficiency due to the formation of an acoustic barrier. Fadimu et al. [23] reported that, under identical ultrasonic conditions (400 W, 10 min), hydrolysates prepared with alcalase exhibited the highest DPPH scavenging and metal-chelating activities. In contrast, those prepared with flavorzyme showed superior hypoglycemic and antihypertensive activities, likely due to differences in proteolytic cleavage patterns. These findings indicate that the efficacy of ultrasound-assisted enzymatic hydrolysis is influenced by multiple factors, including ultrasound power and protease type.

Although previous studies have demonstrated the feasibility of ultrasound-assisted enzymatic hydrolysis for freshwater mussels, they have primarily focused on extraction efficiency and bioactivity [24,25]. However, the relationships among ultrasonic power, protease specificity, and the physicochemical and functional properties of the resulting hydrolysates remain insufficiently understood. Accordingly, this study aims to investigate the effects of pretreatment with different ultrasonic powers (100 W, 200 W, 300 W, 400 W, 500 W, and 600 W) and hydrolysis with different proteases (flavorzyme, alcalase, bromelain, and trypsin) on pearl mussel hydrolysates. First, the effects of combined ultrasonic and protease treatment on the degree of hydrolysis (DH) and molecular weight distribution of the hydrolysate were examined. Second, the structural characteristics, physicochemical properties, and amino acid composition of the hydrolysate were characterized. Finally, the antioxidant activity, hypoglycemic activity, and functional properties of the hydrolysate were evaluated. By integrating these findings, the potential mechanisms linking ultrasonication and protease treatment to the physicochemical and functional properties of the pearl mussel hydrolysate were comprehensively analyzed. The results of this study are expected to advance the utilization of aquatic byproducts in food applications and provide valuable insights for the development of functional foods.

## 2. Results

### 2.1. Effects of Different Ultrasonic Power and Proteases on the DH and TCA Soluble Protein Content of Pearl Mussel Hydrolysates

**Figure 1:**
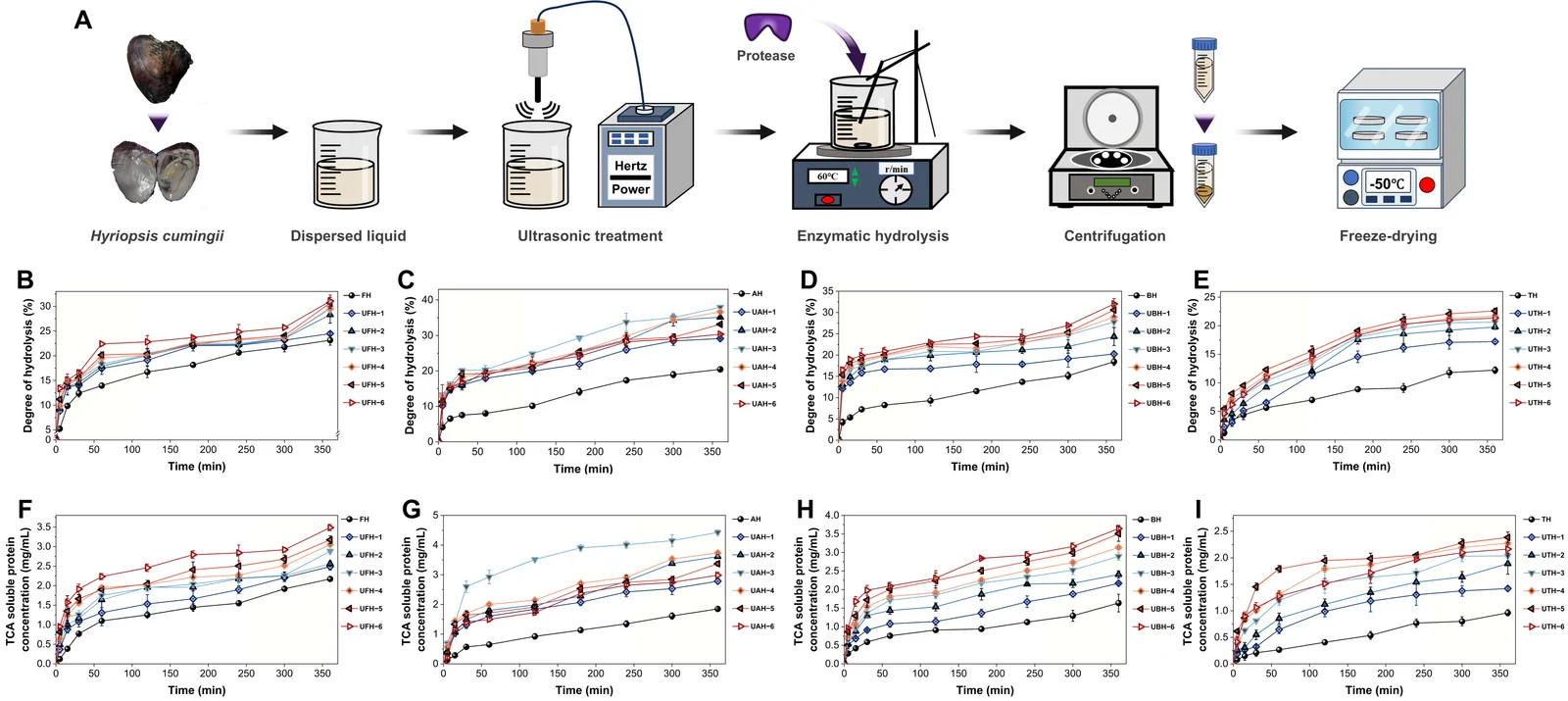
Process for preparing pearl mussel hydrolysate (**A**), degree of hydrolysis (**B–E**), and TCA-soluble protein content (**F–I**) of pearl mussel hydrolysates under different ultrasound power and protease treatment conditions.

As shown in Figure 1, the degrees of hydrolysis (DH) and TCA soluble protein content of the hydrolysates in each group gradually increased over time, reaching a maximum at 360 min. Among the enzymes tested, the alcalase group exhibited higher DH (20.43–37.93%) and TCA soluble protein contents (1.85–4.42 mg/mL). The bromelain group demonstrated superior hydrolytic activity than the flavorzyme group, whereas the trypsin group showed significantly lower hydrolytic activity than the other groups (*p* < 0.05). Alcalase has a broad range of cleavage sites, primarily targeting peptide bonds containing aromatic amino acids. In contrast, trypsin primarily acts on C-terminal peptide bonds of lysine and arginine residues [26]. Magalhães et al. [27] reported that alcalase exhibited significantly higher hydrolytic activity toward goat milk casein than flavorzyme, consistent with our findings. In contrast, another study reported that trypsin exhibited superior hydrolytic activity toward hempseed protein compared with flavorzyme and bromelain; this may be attributed to differences in raw material type, enzyme-to-substrate ratio, and reaction temperature [5].

The shear forces, pressure fluctuations, and temperature variations generated by ultrasonic treatment can induce structural changes in the substrate, significantly enhancing its binding affinity with proteases [19]. The results of this study show that the DH of different protease groups was significantly higher after ultrasonic treatment compared to the untreated group (*p* < 0.05). Figure 1B–E illustrate that the DH of the flavorzyme and the trypsin group increased with increasing ultrasonic power (100–600 W), reaching a maximum of 31.05% and 32.02%, respectively, at 600 W. The cavitation effect generated by ultrasound induces random substrate cleavage via strong shear forces. It promotes the conversion of water molecules into free radicals, thereby altering substrate structure and enhancing enzymatic hydrolysis efficiency [28]. In contrast, the hydrolysis rates of the alcalase and the trypsin group first increased and then decreased with increasing ultrasonic power, peaking at 300 W (37.93%) and 500 W (22.60%), respectively. This pattern may be attributed to several factors: (I) high ultrasonic power tends to induce protein aggregation, masking enzyme cleavage sites [29]; (II) high ultrasonic power promotes exposure of hydrophobic groups, reducing protein–water interactions [28]; and (III) bubble clusters formed at high power shield smaller bubbles near solutes, weakening the cavitation effect [19]. Notably, ultrasonic treatment increased the DH of the flavorzyme group by only 34.13%, which was significantly lower than that of the other groups (73.6–85.64%, *p* < 0.05). Davoudi et al. [20] reported that ultrasonic pretreatment enhanced the hydrolytic capacity of pepsin toward zein but had no significant effect on enzymatic hydrolysis by papain. These findings indicate that ultrasonic-induced structural changes in proteins have differential effects on cleavage efficiency for different proteases, a phenomenon also observed in the present study.

The results of this study indicate that, in terms of hydrolytic activity, alcalase demonstrates considerable potential for commercial applications. Moreover, ultrasonic power is also a critical factor in influencing hydrolytic performance.

### 2.2. Effects of Different Ultrasonic Power and Proteases on the Particle Size and ζ-Potential of Pearl Mussel Hydrolysates

**Figure 2:**
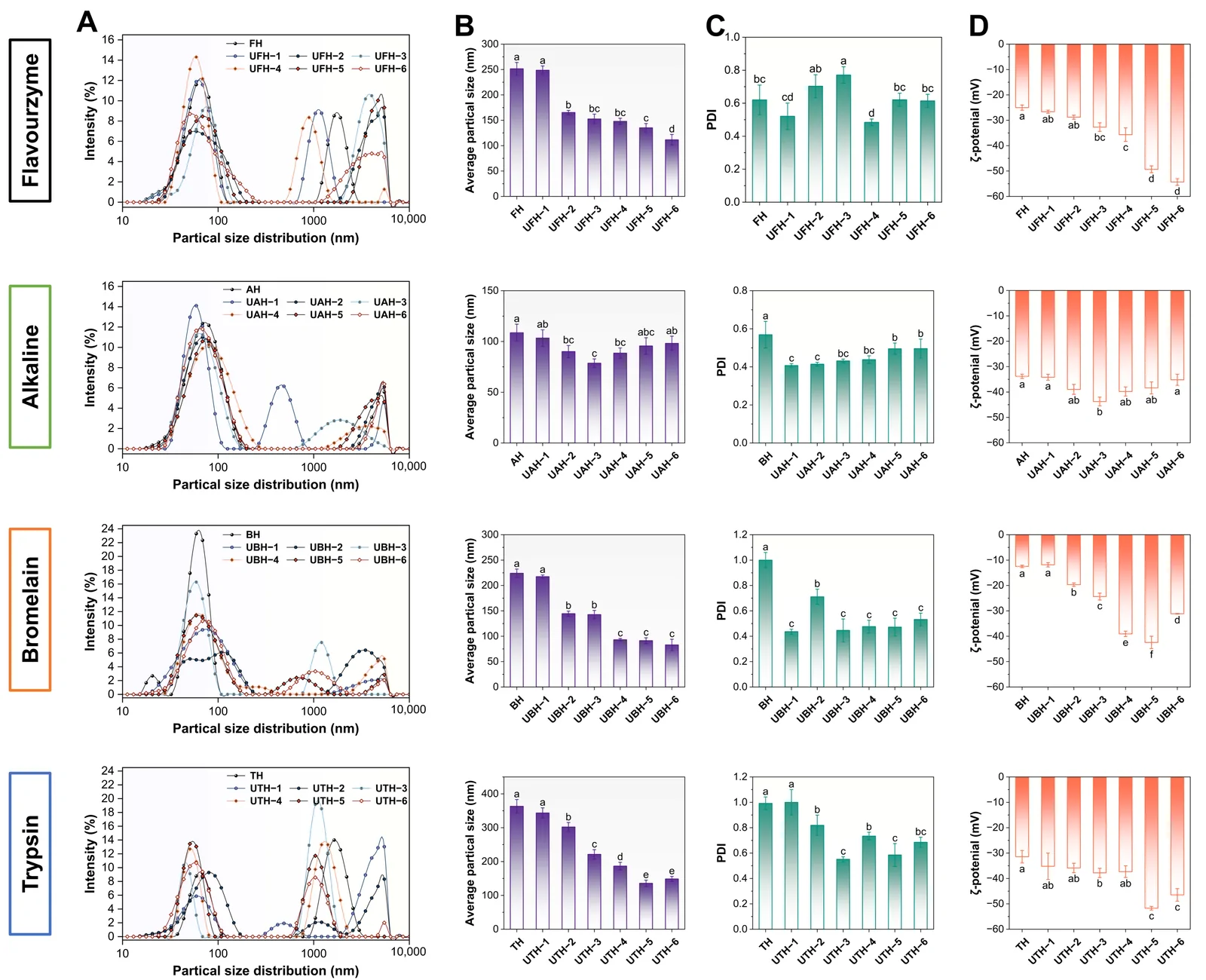
Particle size distribution (**A**), average particle size (**B**), PDI (**C**), and ζ-potential (**D**) of pearl mussel hydrolysates under different ultrasonic power and protease treatment conditions. Different letters indicate significant differences among groups (*p* < 0.05), whereas groups sharing the same letter are not significantly different (*p* > 0.05).

The particle size distribution results for the hydrolysates in each group are shown in Figure 2A. Without ultrasonic treatment, the particle size distributions of the pearl mussel hydrolysates prepared with the four proteases were as follows: FH 29–2990 nm, AH 236–6358 nm, BH 15–895 nm, and TH 32–3477 nm. All groups exhibited a multi-peak distribution, consistent with the findings of Liu et al. [30] for mung bean protein hydrolysates, where enzymatic hydrolysis broadens the particle size distribution. Following ultrasonic pretreatment, the UAH-3 group displayed particle size ranges of 23–169 nm and 568–4731 nm, whereas the UTH-5 group exhibited a larger peak area in the 27–92 nm range.

The average particle sizes of the FH, AH, BH, and TH groups were 254.47 nm, 108.52 nm, 224.10 nm, and 363.26 nm, respectively. Among these, the alcalase group exhibited the smallest particle size, while the trypsin group had the largest, consistent with the DH results. Du et al. [31] reported that alcalase hydrolyzed goat milk casein into shorter peptides, resulting in a significantly greater reduction in particle size than other protease groups. Ultrasonic treatment reduced the particle size of hydrolysates in all groups. High-power ultrasonics significantly decreased the particle size in the flavorzyme, bromelain, and trypsin groups (UFH-6: 111.57 nm, UBH-6: 82.88 nm, UTH-5: 135.78 nm; *p* < 0.05). In the alcalase group, particle size decreased most significantly at moderate ultrasonic power (300 W), reaching 78.61 nm. Ultrasonication disrupts hydrogen bonds, hydrophobic interactions, and electrostatic interactions among proteins or peptides, thereby increasing DH and promoting the formation of small-particle-size products [32].

The polydispersity index (PDI) is commonly used to characterize the molecular weight distribution of hydrolysates, with lower values indicating a more uniform distribution. Without ultrasonic treatment (Figure 2C), the PDI of the bromelain and trypsin groups was significantly higher than that of the flavorzyme group (0.65) and the alcalase group (0.57), indicating that the alcalase group had a more uniform molecular weight distribution. Duan et al. [6] reported that alcalase hydrolysates of bovine liver protein with higher DH exhibited lower PDI. Higher-intensity ultrasonic pretreatment significantly reduced the PDI of hydrolysates in all groups, enhancing their dispersion, particularly in the bromelain group (41.50% decrease) and trypsin group (55.40% decrease).

Higher absolute values of the ζ-potential generally indicate stronger electrostatic repulsion, which can effectively inhibit protein or peptide aggregation and improve solution dispersion. Without ultrasonic treatment (Figure 2D), the ζ-potentials of FH, AH, BH, and TH were −25.01 mV, −33.81 mV, −12.48 mV, and −31.48 mV, respectively. Among these, the alcalase group exhibited a significantly higher absolute ζ-potential than the other groups (*p* < 0.05), indicating excellent dispersibility in water and suggesting potential functional advantages. Liu et al. [30] reported that mung bean protein hydrolysates prepared with alcalase showed the highest absolute ζ-potential, consistent with our findings. Ultrasonic pretreatment further increased the absolute ζ-potential values in all groups. Notably, the flavorzyme group exhibited a significant increase in absolute surface charge (−55.32 mV) after ultrasonic treatment at 600 W (*p* < 0.05). Ultrasonication promotes protein unfolding and inhibits protein refolding or aggregation. Yang et al. [33] found that ultrasonic-assisted enzymatic hydrolysis enhanced the absolute ζ-potential of housefly (*Musca domestica*) larval protein hydrolysate, improving its solubility and emulsification properties.

### 2.3. Effects of Different Ultrasonic Power and Proteases on the UV and Fluorescence Spectra of Pearl Mussel Hydrolysates

**Figure 3:**
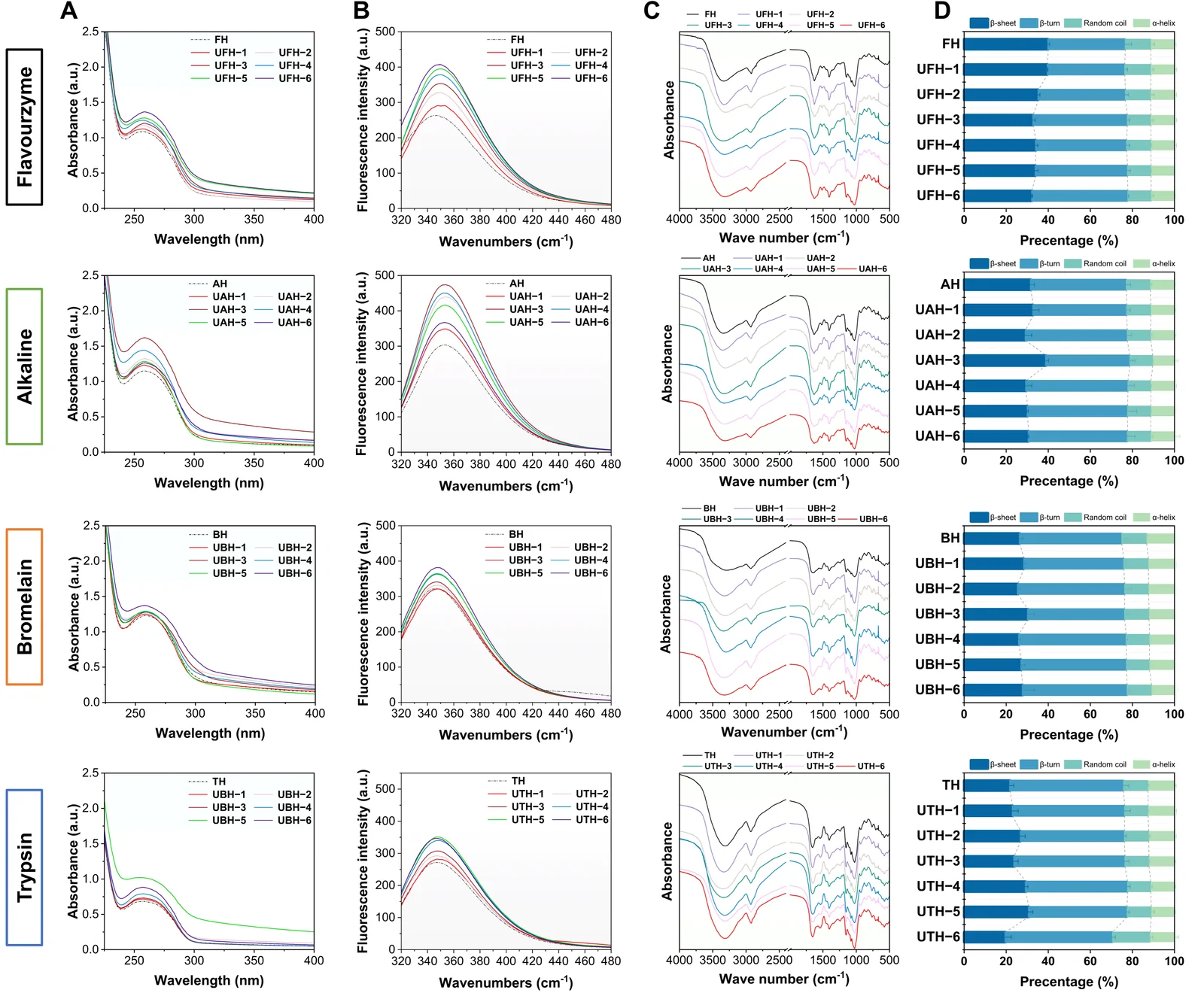
UV spectra (**A**), fluorescence spectra (**B**), FTIR (**C**), and secondary structures (**D**) of pearl mussel hydrolysates under different ultrasonic power and protease treatment conditions.

UV spectra are primarily influenced by aromatic amino acids (phenylalanine, tryptophan, and tyrosine) and are commonly used to rapidly detect changes in protein structure [14]. All hydrolysates exhibited absorption peaks at 250–300 nm (Figure 3A). Without ultrasonic pretreatment, the alcalase and bromelain groups showed higher UV absorption intensities than the other groups, likely due to differences in protease cleavage specificity. The broad specificity of alcalase allows it to release more aromatic amino acids, thereby increasing UV intensity [11]. Ultrasonic pretreatment further enhanced the UV absorption of hydrolysates in all groups, with the most pronounced increase observed in the alcalase group (UAH-3: 1.61, *p* < 0.05). Quan et al. [19] reported that ultrasonic treatment significantly enhanced the UV intensity of oyster peptides, as cavitation and mechanical effects promoted protein–protein interactions and exposed more hydrophobic amino acids.

Fluorescence spectra reflect changes in the microenvironment of aromatic amino acids as a function of excitation wavelength. They are highly sensitive, making them useful for assessing protein tertiary structure [5]. The fluorescence spectra of the hydrolysates from each group are shown in Figure 3B. Without ultrasonic treatment, the order of maximum fluorescence intensity was BH > AH > FH > TH. Duan et al. [6] reported that bovine liver hydrolysates prepared with alcalase exhibited higher fluorescence intensities. In contrast, another study found that bromelain treatment reduced the fluorescence intensity of goat milk whey protein, which differs from the results of the present study, suggesting that raw material type can influence the exposure of aromatic groups after hydrolysis [31].

After ultrasonic treatment, the maximum fluorescence intensities for flavorzyme, alcalase, bromelain, and trypsin were 406 a.u. (UFH-6), 474 a.u. (UAH-3), 381 a.u. (UBH-6), and 351 a.u. (UTB-5), respectively. Which were significantly higher than those of the non-ultrasonicated group (*p* < 0.05). Similar findings have been reported for Acheta domesticus protein hydrolysates, in which ultrasonication induced conformational changes in substrate proteins, thereby promoting the exposure of internal fluorescence-sensitive groups [22].

### 2.4. Effects of Different Ultrasonic Power and Proteases on the FTIR of Pearl Mussel Hydrolysates

FTIR is a molecular vibrational technique commonly used to characterize functional groups and the secondary structure of proteins [33]. The infrared spectra of the hydrolysates in the 4000–400 cm^−1^ range are shown in Figure 3C. The signal peak near 3400–3200 cm^−1^ corresponds to amide A, arising from N–H bending and O–H stretching vibrations, and indicates the presence of hydrogen bond interactions between peptide chains. The hydrolysates in each group exhibited differences in peak intensity and width in the amide A region, reflecting the varying effects of proteases on hydrogen bonding [6]. Ultrasonic treatment enhanced the intensity of the amide A peak, as cavitation-induced intermolecular rearrangements strengthened hydrogen bonds in the hydrolysates [22]. The signal peak near 2930 cm^−1^ is attributed to the asymmetric stretching of C–H in fatty acid side chains, suggesting that ultrasonic-assisted hydrolysis disrupted hydrophobic regions of the proteins and altered the microenvironment of alkyl chains [30]. Peaks near 1643 cm^−1^ and 1520 cm^−1^ correspond to amide I and amide II, representing C=O stretching, N–H bending, and C–N stretching, respectively, while the absorption peak near 1253 cm^−1^ corresponds to amide III [34]. Following ultrasonic treatment, amide I shifted to higher wavenumbers (1624–1645 cm^−1^), consistent with observations by Ballon et al. [16] in the lesser mealworm (*Alphitobius diaperinus larva*) protein hydrolysate, indicating a change in the conformation of the protein main chain.

Different regions of the amide I band correspond to specific protein secondary structures: 1700–1660 cm^−1^ (β-turn), 1660–1650 cm^−1^ (α-helix), 1650–1640 cm^−1^ (random coil), and 1640–1600 cm^−1^ (β-sheet). By applying deconvolution and Gaussian filtering to the amide I spectrum, the content of secondary structures in each hydrolysate was calculated. As shown in Figure 3D, the hydrolysates were predominantly composed of β-turns and β-sheets. The flavorzyme group exhibited the highest β-sheet content (32.25–39.45%), the bromelain group had the highest β-turn content (46.03–50.96%), and the alcalase group showed the lowest α-helix (10.31–11.49%) and random coil (10.90–11.44%) content. Du et al. [31] reported that goat milk whey protein hydrolysates prepared with alcalase exhibited lower α-helix and random coil content, consistent with our findings. The reduction in α-helix content indicates that proteases disrupted hydrogen bonds in the protein, leading to a transition from compact α-helix structure to β-turns, which can enhance molecular flexibility [30]. Ultrasonic pretreatment increased the β-turn content in the flavorzyme and trypsin groups, whereas it increased the β-sheet content in the alcalase and bromelain groups. This effect is attributed to cavitation-induced disruption of protein structures. The β-turn structure is associated with hydrophilicity, and increased hydrophilicity enhances the stability and bioactivity of hydrolysates [33]. Overall, the combination of ultrasonication and enzymatic hydrolysis promotes the formation of more unfolded structures. However, the precise contribution of these structural changes to biological activity and functionality remains to be clarified.

### 2.5. Effects of Different Ultrasonic Power and Proteases on the Surface Hydrophobicity and Total Sulfhydryl Content of Pearl Mussel Hydrolysates

**Figure 4:**
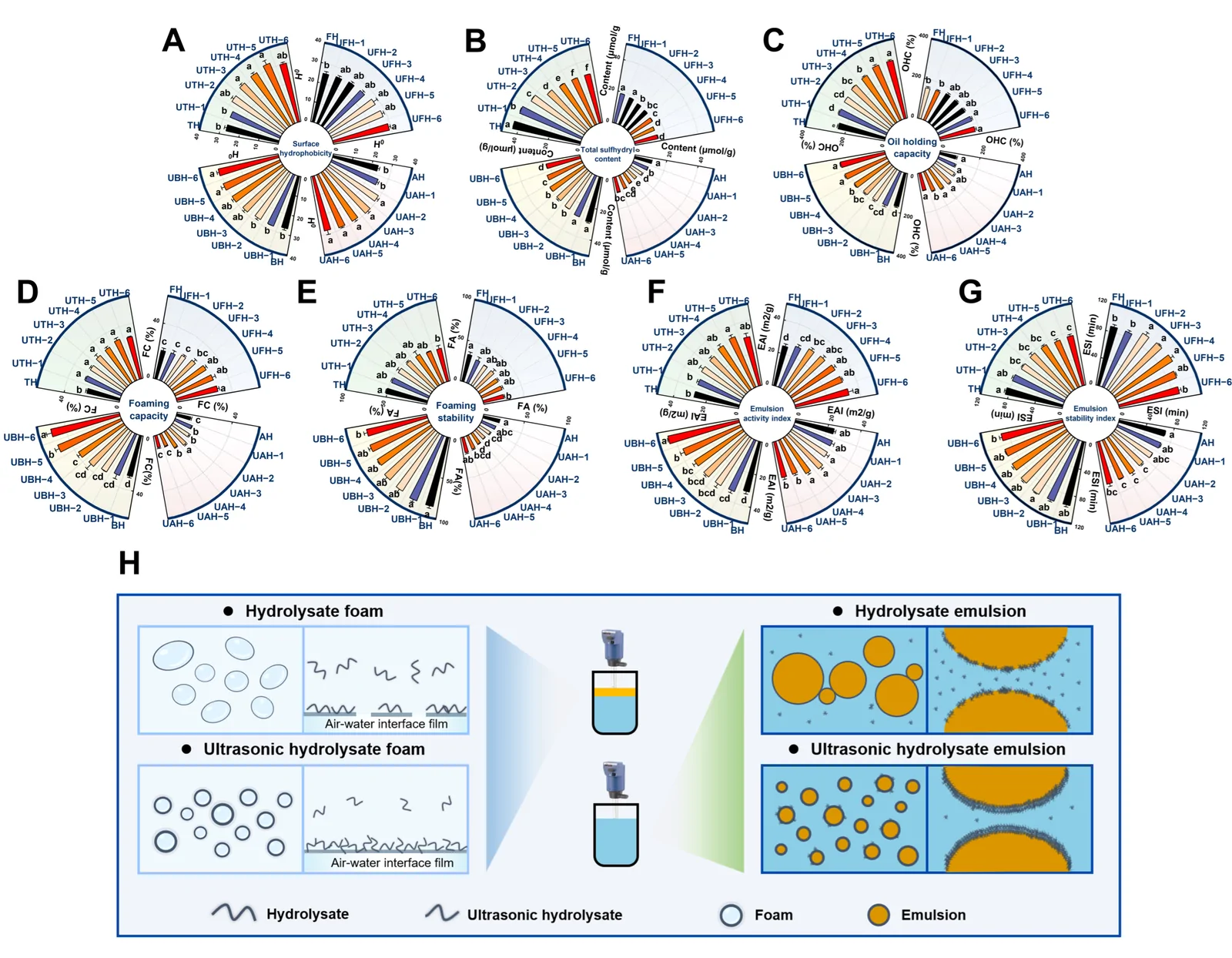
Surface hydrophobicity (**A**), total sulfhydryl content (**B**), oil holding capacity (**C**), foaming capacity (**D**), foaming stability (**E**), emulsion activity (**F**), emulsion stability (**G**), and foam and emulsion mechanisms (**H**) of pearl mussel hydrolysates under different ultrasonic power and protease treatment conditions. Different letters indicate significant differences among groups (*p* < 0.05), whereas groups sharing the same letter are not significantly different (*p* > 0.05).

The surface hydrophobicity of the hydrolysates from each group is shown in Figure 4A. Without ultrasonic treatment, the flavorzyme group (24.87) exhibited lower surface hydrophobicity, whereas the trypsin group showed the highest value (26.90). This observation is consistent with Tan et al. [35], who reported that trypsin treatment exposes more hydrophobic amino acids in soy protein. Ultrasonic pretreatment increased the surface hydrophobicity of the hydrolysates across all groups by exposing hidden hydrophobic sites within the proteins, thereby facilitating proteolytic cleavage and the formation of additional hydrophobic amino acid residues [21]. Notably, the effect of ultrasonic power on surface hydrophobicity differed among protease groups: flavorzyme and bromelain showed a gradual increase with increasing power, whereas alcalase and trypsin exhibited an initial increase followed by a decline. This pattern may be attributed to the ultrasonic power-dependent behavior of pearl mussel hydrolysates, as demonstrated in studies on Asian bullfrog collagen hydrolysates, in which excessive ultrasonic power can induce protein aggregation, thereby reducing surface hydrophobicity [36].

Total sulfhydryl content is an important indicator of protein stability and functionality. Figure 4B shows the total sulfhydryl content of the hydrolysates from each group. Without ultrasonic treatment, the total sulfhydryl contents of FH, AH, BH, and TH were 18.71, 11.00, 28.97, and 43.31 μmol/g, respectively. Protease cleavage of peptide bonds induces conformational changes in proteins, leading to the loss or modification of some thiol-containing peptides and, consequently, decreasing total sulfhydryl content [37]. Among them, AH, which exhibited the highest DH, had the lowest total sulfhydryl content. This finding is consistent with Wang et al. [5], who reported that hempseed protein hydrolysates with lower DH typically exhibit higher total sulfhydryl content. The observed differences in sulfhydryl content among protease groups may be attributed to several factors: (I) hydrolysis temperature and pH influence the extent of sulfhydryl reduction; (II) protease cleavage specificity leads to variations in the exposure of protein thiols [38]. Ultrasonic treatment significantly reduced total sulfhydryl content in all hydrolysates. The shear and vortex forces generated by ultrasound induce structural changes, creating additional cleavage sites and increasing the likelihood of thiols being oxidized to form disulfide bonds (S–S) or other sulfur-containing derivatives, such as sulfonic acids and sulfites [39].

### 2.6. Effects of Different Ultrasonic Power and Proteases on the SEM of Pearl Mussel Hydrolysates

**Figure 5:**
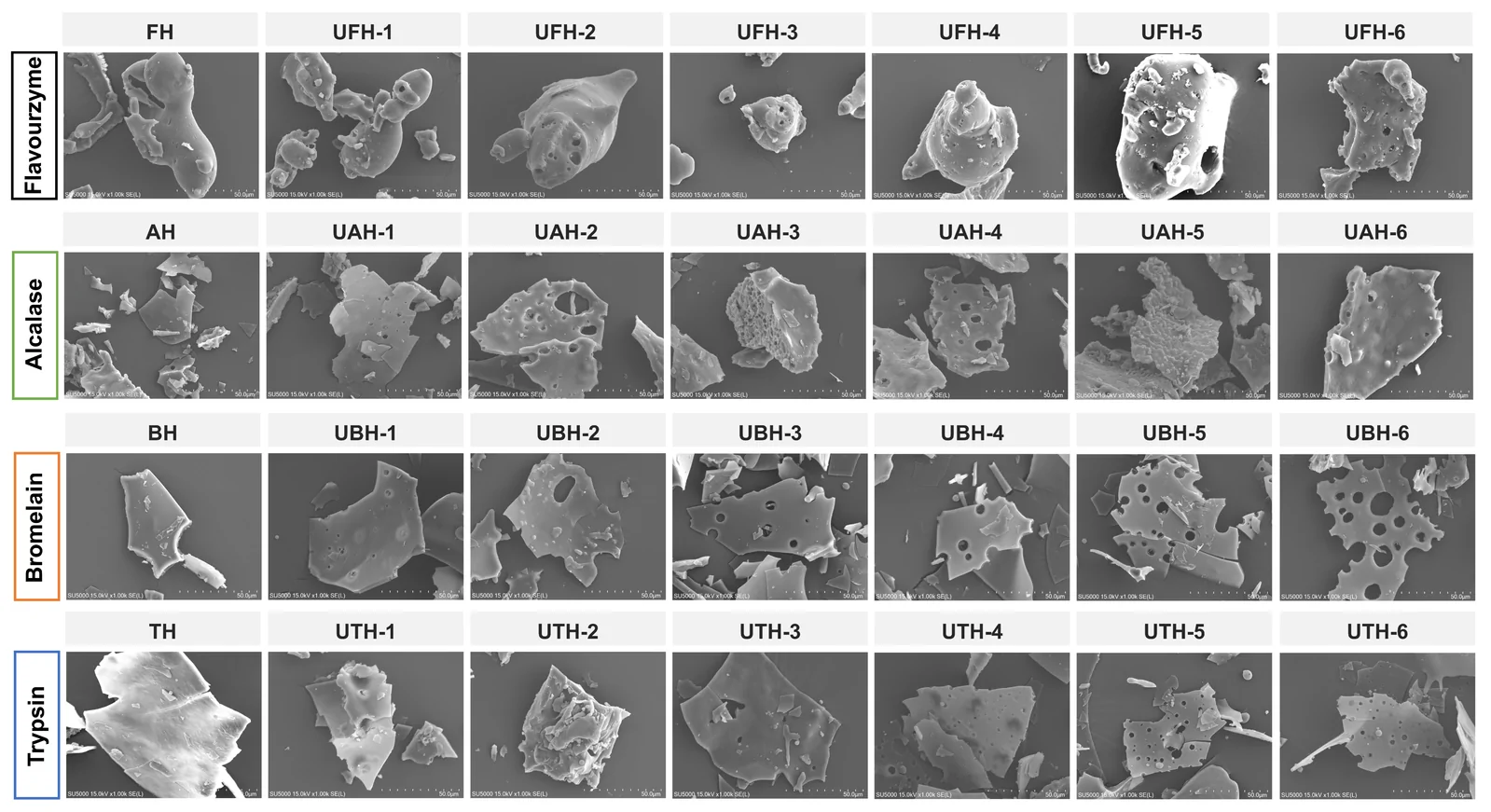
SEM of pearl mussel hydrolysates under different ultrasonic power and protease treatment conditions. Magnification: 1000×.

The microstructures of the hydrolysates were observed using SEM at 1000× magnification (Figure 5). Without ultrasonic treatment, the hydrolysates in each group exhibited distinct microstructural characteristics: the FH group showed a smooth surface with irregular oval-shaped particles, whereas the AH, BH, and TH groups displayed smooth surfaces with irregular flake-like structures. The small fragmentary particles observed on the surfaces of some hydrolysates may be attributed to the re-aggregation of small peptides generated during enzymatic hydrolysis [5]. After ultrasonic treatment, pores formed on the surfaces of the hydrolysates in all groups. The alcalase and bromelain groups exhibited larger pores, whereas the flavorzyme and trypsin groups primarily displayed smaller pores. It has been reported that porous and loose structures facilitate interactions between hydrolysates and water, thereby enhancing solubility and antioxidant activity [33]. Similar observations have been reported for zein peptides [14] and walnut peptides [40]. The microjets generated by ultrasonication disrupt the internal compact structure of proteins, thereby increasing their surface area and the number of cleavage sites. These results indicate that ultrasonic treatment positively enhances enzymatic hydrolysis.

### 2.7. Effects of Different Ultrasonic Power and Proteases on the Amino Acid Composition of Pearl Mussel Hydrolysates

**Figure 6:**
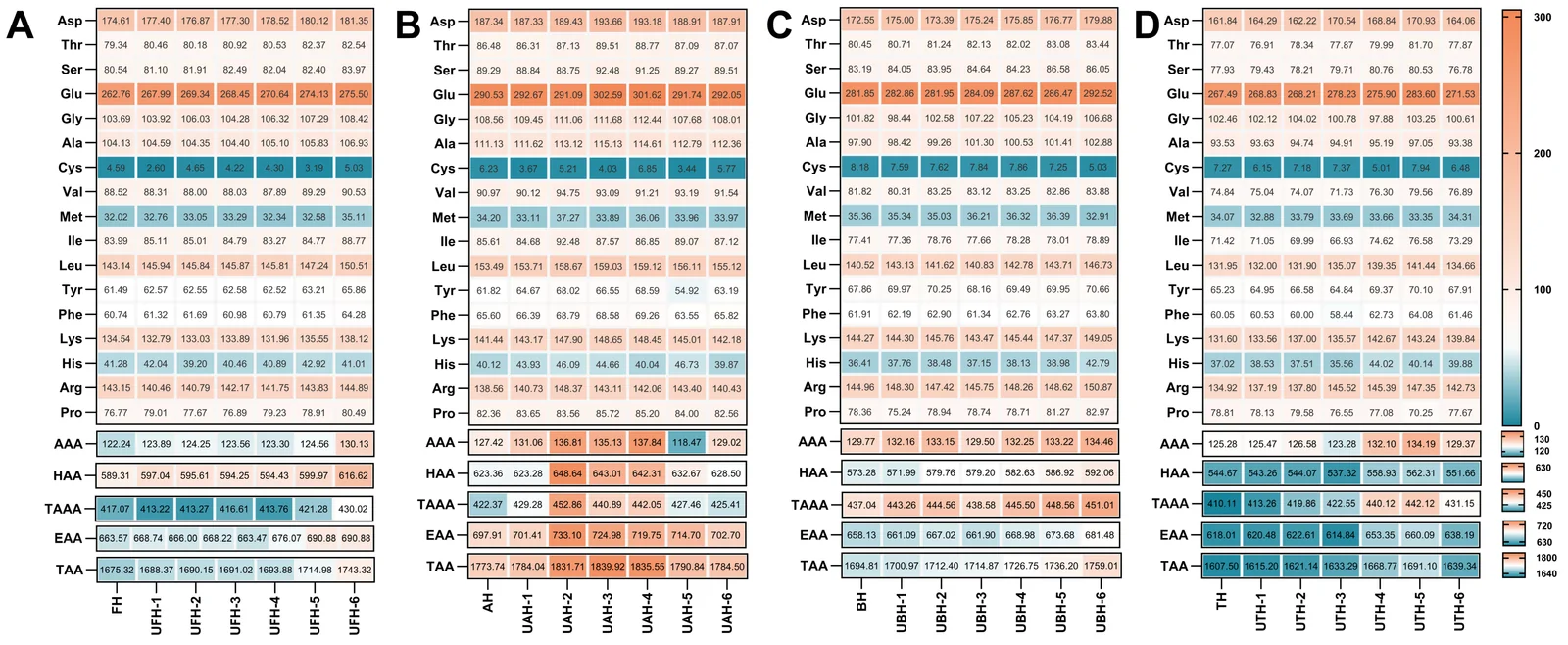
Amino acid composition (mg/100 g sample) of pearl mussel hydrolysates under different ultrasonic power and protease treatment conditions: flavorzyme (**A**), alcalase (**B**), bromelain (**C**), and trypsin (**D**). Note: Asp: aspartic acid; Thr: threonine; Ser: serine; Glu: glutamic acid; Gly: glycine; Ala: alanine; Cys: cysteine; Val: valine; Met: methionine; Ile: isoleucine; Leu: leucine; Tyr: tyrosine; Phe: phenylalanine; Lys: lysine; His: histidine; Arg: arginine; Pro: proline. AAA: sum of the quantified aromatic amino acids (Tyr and Phe); HAA: sum of the quantified hydrophobic amino acids (Ala, Pro, Val, Ile, Met, Leu, and Phe); TAAA: sum of the quantified antioxidant associated amino acids (Cys, Met, Tyr, His, Lys, and Arg); EAA: sum of the quantified essential amino acids (Thr, Val, Met, Ile, Leu, Phe, His, and Lys); TAA: total quantified amino acids.

Figure 6 shows the amino acid composition of the hydrolysates from each group, with a total of 17 amino acids detected in all samples. Following treatment with different ultrasonic powers and proteases, Glu (262.76–302.59 mg/100 g), Asp (161.84–193.66 mg/100 g), Leu (140.52–159.12 mg/100 g), Lys (131.60–149.05 mg/100 g), Arg (134.92–150.87 mg/100 g), Ala (93.38–115.13 mg/100 g), and Gly (97.88–112.44 mg/100 g) were identified as the predominant amino acids across all groups. Notably, the alcalase group exhibited the highest levels of AAA (137.84 mg/100 g), HAA (648.64 mg/100 g), TAAA (452.86 mg/100 g), EAA (733.10 mg/100 g), and TAA (1839.92 mg/100 g) (Table S1). This may be attributed to alcalase’s strong hydrolytic capacity and its specificity for amino acids such as Tyr, Met, and Phe. AAA is associated with enhanced free radical scavenging activity [41]; HAA contributes to hydrolysate stability and serves as an important indicator for emulsifying properties [42]; EAA reflects the potential of pearl mussel hydrolysates as dietary protein supplements [43]; and TAAA is positively correlated with the antioxidant activity [44,45]. Recent studies have further indicated that hydrolysates rich in AAA, HAA, and EAA promote protein aggregation, thereby enhancing gastrointestinal adsorption and exerting beneficial biological activities [46].

Ultrasonic treatment significantly improved the amino acid profiles of hydrolysates in all groups. The TAA of the four hydrolysates increased by 4.06% (UFH-6), 3.73% (UAH-3), 3.79% (UBH-6), and 5.20% (UTH-5), respectively. This finding is consistent with Ding et al. [14], who demonstrated that ultrasonic-assisted enzymatic hydrolysis significantly increased the TAA content of zein hydrolysates. The cavitation effect of ultrasound induces structural changes in proteins, leading to the formation of smaller particles and an increase in substrate surface area. This enhances enzyme–substrate interactions, thereby promoting proteolysis and increasing TAA content in the hydrolysates [21]. Additionally, ultrasonic treatment significantly increased the contents of Asp, Thr, Glu, Lys, Tyr, and Phe. Fadimu et al. [23] reported that ultrasonic treatment of lupin protein hydrolysates increased the Asp and Thr contents; after 10 min of treatment, the alcalase hydrolysate exhibited a lower DPPH scavenging activity IC_50_ value (3.77 mg/mL) compared to other groups. Admassu et al. [47] found that peptides containing Glu and Lys in their side chains exhibited stronger α-amylase inhibitory activity, mediated through interactions with enzyme residues and steric hindrance. Furthermore, peptides containing Tyr and Phe at the C- and N-termini, respectively, exhibit stronger interactions with angiotensin-converting enzyme (ACE), thereby enhancing ACE-inhibitory activity [48].

In summary, pearl mussel hydrolysates exhibit potential as a novel protein supplement and functional food ingredient.

### 2.8. Effects of Different Ultrasonic Power and Proteases on the Biological Activity of Pearl Mussel Hydrolysates

#### 2.8.1. Antioxidant Activity

Given the limitations of relying on a single indicator, this study employed four indicators (DPPH radical scavenging activity, ABTS radical scavenging activity, hydroxyl radical scavenging activity, and reducing capacity) to evaluate the antioxidant activity of pearl mussel hydrolysates systematically.

**Figure 7:**
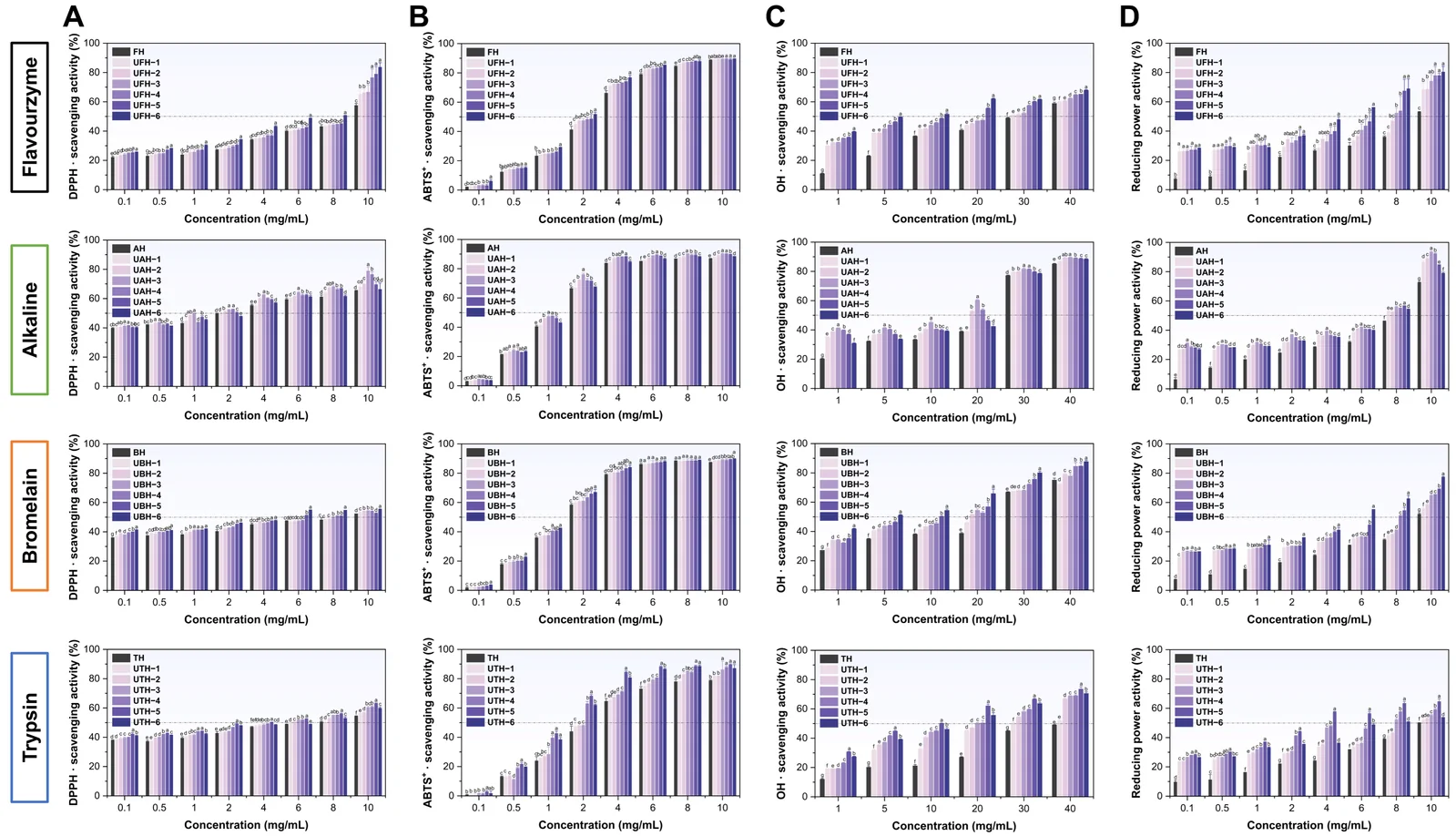
DPPH radical scavenging activity (**A**), ABTS radical scavenging activity (**B**), hydroxyl radical scavenging activity (**C**), and reducing power (**D**) of pearl mussel hydrolysates under different ultrasonic power and protease treatment conditions. Different letters indicate significant differences among groups (*p* < 0.05), whereas groups sharing the same letter are not significantly different (*p* > 0.05).

DPPH is an unstable free radical whose absorbance decreases upon contact with a hydrogen donor. Without ultrasonic treatment, the DPPH radical scavenging activity of the four protease hydrolysates exhibited a dose-dependent relationship (Figure 7A), with the alcalase hydrolysate (AH) showing significantly higher activity (65.61%) than the other groups (*p* < 0.05). Similar results were reported by Jin et al. [38], who found that the highly hydrolyzed *Pseudosciaena crocea* roe hydrolysate (PCRH-120) exhibited stronger DPPH radical scavenging activity. The broad hydrolytic specificity of alcalase enables the generation of short-chain peptides with high electron density and redox potential, which can effectively scavenge free radicals by donating electrons [49].

ABTS is a chromophore compound that can be reduced to a colorless form by antioxidants and is commonly used to evaluate the antioxidant activity of hydrolysates. The ABTS radical scavenging activity of the four protease hydrolysates was stabilized at concentrations of 6–10 mg/mL (Figure 7B), with the activities of the FH, AH, and BH groups being significantly higher than that of the TH group. This difference may be attributed to variations in the content of aromatic and hydrophobic amino acids. The hydroxyphenyl group of Tyr can form stable compounds with radicals via resonance interactions, while hydrophobic residues enhance interactions between hydrolysates and hydrophobic radicals [6]. Furthermore, peptides with molecular weights < 3 kDa from the pearl shell (*Pinctada martensii*) exhibit significantly higher ABTS radical scavenging activity than crude peptides, emphasizing the stronger antioxidant activity of low-molecular-weight peptides. Thus, the influence of peptide molecular weight on antioxidant activity should not be overlooked [9].

Hydroxyl radicals readily react with biomacromolecules, causing physiological disturbances in the body. The hydroxyl radical scavenging activity of the four protease hydrolysates is presented in Figure 7C. Among the hydrolysates, AH exhibited the strongest scavenging activity, while BH showed significantly higher activity than FH at elevated concentrations. Previous studies generally indicate a positive correlation between DH and antioxidant activity. Hydrolysis releases more active amino acids and short peptides, thereby enhancing scavenging capacity; however, excessive hydrolysis may reburial active groups, reducing activity [11,46]. Notably, BH contains higher levels of AAA and TAA than FH, which may contribute significantly to its antioxidant activity. In general, peptides with terminal residues such as Met, Tyr, Cys, and Lys can efficiently donate electrons to free radicals, thereby terminating radical chain reactions [38].

Reducing power is a widely used indicator for evaluating a substance’s electron-donating ability. As the concentration increased, the reducing power of the four protease hydrolysates gradually rose, with FH, AH, BH, and TH showing increases of 5.89-, 9.88-, 6.00-, and 3.99-fold, respectively. Among them, AH exhibited the highest reducing power (72.73%), significantly surpassing the other groups, indicating its potential antioxidant capacity. Similar findings have been reported for bovine liver hydrolysates, in which alcalase-treated hydrolysates exhibited stronger reducing power, and the release of antioxidant amino acids during hydrolysis enhanced their ability to disrupt free radical chains [6]. Additionally, other studies have suggested that specific amino acids, such as Glu, Gly, and Met, contribute positively to reducing power [23]. These observations are consistent with our results, as higher levels of active amino acids were detected in the AH group.

Ultrasonic treatment effectively enhanced the antioxidant activity of the hydrolysates. As ultrasonic power increased, the antioxidant activity of the flavorzyme and bromelain groups gradually rose. In contrast, the alcalase and trypsin groups exhibited an initial increase followed by a decrease, consistent with the observed trends in DH. Davoudi et al. [20] reported that among zein hydrolysates prepared using three proteases (alcalase, papain, and pepsin), the alcalase hydrolysate with the highest DH (16.62%) demonstrated significantly higher antioxidant activity than the other groups (*p* < 0.05). Similarly, Quan et al. [19] observed that oyster peptides subjected to a 200 W ultrasonic treatment exhibited the highest soluble peptide content and antioxidant capacity among the groups. These findings indicate a positive correlation between DH and antioxidant activity [20]. Moreover, ultrasonic power plays a critical role in regulating the antioxidant activity of hydrolysates. Hao et al. [18] found that the ABTS radical scavenging activity of porcine bone collagen peptides peaked at 300 W, whereas treatment at 450 W decreased activity. Appropriate ultrasonic treatment can induce favorable structural changes in proteins; increase the accessibility of cleavage sites; and promote the release of aromatic amino acids, hydrophobic groups, and low-molecular-weight peptides [20,22]. Conversely, excessive ultrasonic power can generate acoustic barriers, reduce microjet efficiency, and promote protein aggregation, thereby inhibiting the formation of hydrogen donors such as Tyr, Pro, and Ala [18].

The enhancement of hydrolysate antioxidant activity by ultrasound is closely related to structural characteristics, molecular weight, hydrophobicity, and amino acid composition. The shear forces and instantaneous high pressure generated by ultrasound disrupt intermolecular forces within proteins (hydrogen bonds and hydrophobic interactions), thereby altering their secondary and tertiary structures and promoting protein denaturation and fragmentation [28]. Smaller particle sizes and molecular weights facilitate the exposure of active sites, thereby enhancing the antioxidant activity of hydrolysates [20]. Additionally, ultrasound can promote the exposure of hydrophobic groups, improving the accessibility of proteases to peptide regions and releasing more antioxidant peptides [50]. Amino acid composition also plays a critical role. Arg and Glu exhibit strong metal ion-chelating activity. Tyr and Phe enhance radical scavenging activity through electron transfer, and Met and Ala contribute to anti-lipid peroxidation activity [49].

Overall, the UAH-3 exhibited superior antioxidant activity among the pearl mussel hydrolysates. Nevertheless, the investigation of other bioactive properties of the hydrolysate remains necessary.

#### 2.8.2. Hypoglycemic Activity

To comprehensively assess the potential biological activity of pearl mussel hydrolysates, their hypoglycemic effects were evaluated. α-Amylase catalyzes the hydrolysis of starch into oligosaccharides, representing a critical initial step in the conversion of starch to glucose [47]. α-Glucosidase is another key enzyme in starch digestion, primarily responsible for cleaving terminal 1,4-glycosidic bonds [23]. Inhibiting or delaying the activity of these enzymes is widely recognized as an effective strategy for regulating postprandial blood glucose levels.

**Figure 8:**
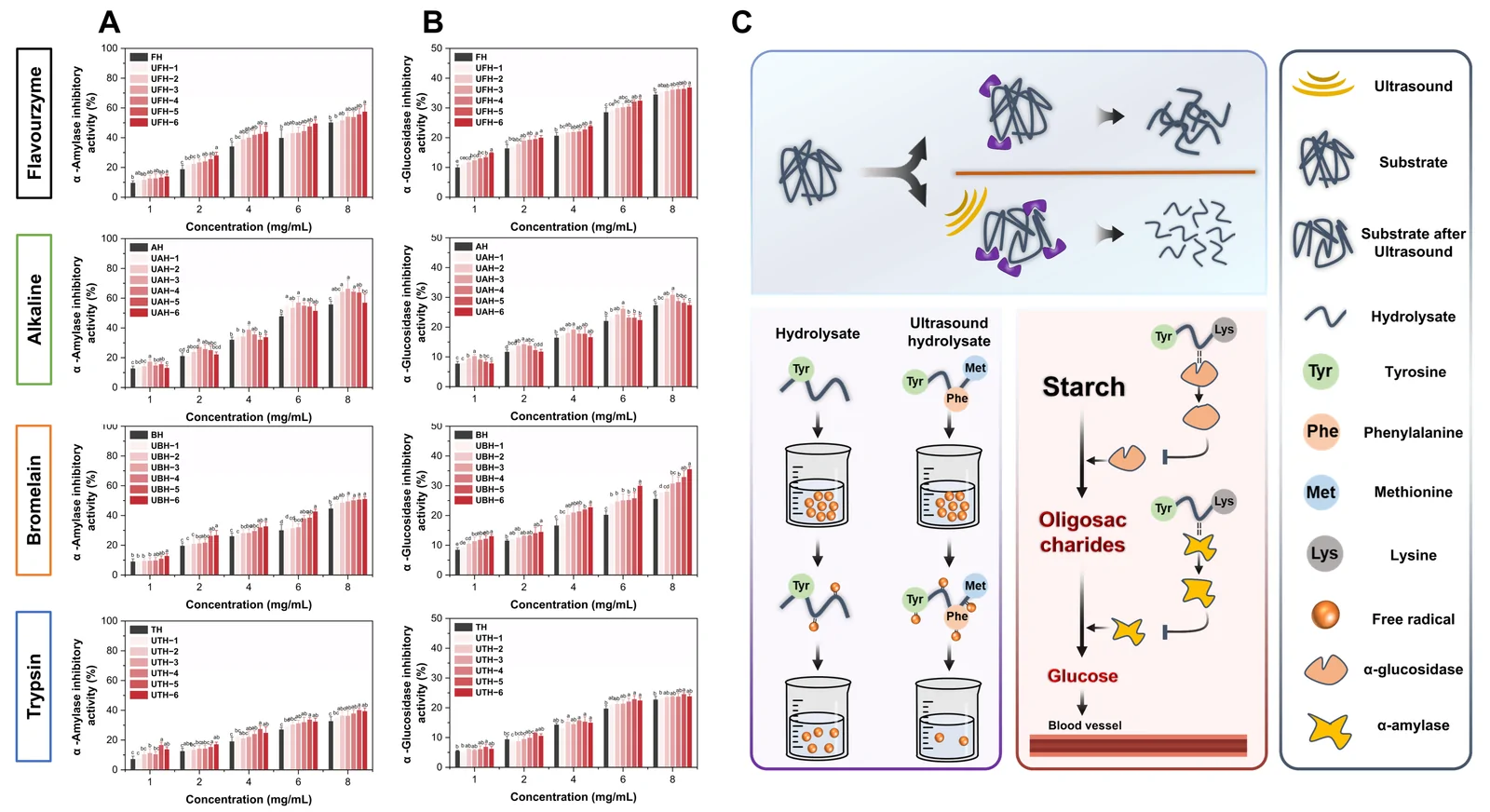
α-amylase inhibitory activity (**A**), α-glucosidase inhibitory activity (**B**), and mechanisms of biological activity (**C**) of pearl mussel hydrolysates under different ultrasonic power and protease treatment conditions. Different letters indicate significant differences among groups (*p* < 0.05), whereas groups sharing the same letter are not significantly different (*p* > 0.05).

The α-amylase inhibitory activities of the hydrolysates from each group are shown in Figure 8A, exhibiting a clear dose-dependent relationship. Without ultrasonic treatment, the inhibitory activity intensities of the four protease hydrolysates (8 mg/mL) were ranked as follows: AH (56.22%) > FH (50.59%) > BH (45.07%) > TH (33.07%). These results are consistent with findings by Du et al. [31], who observed that goat milk whey protein hydrolysates prepared with alcalase demonstrated superior α-amylase inhibitory activity compared to those prepared with bromelain or trypsin, likely due to the lower molecular weight of the alcalase hydrolysates. Moreover, AH contained higher levels of Lys, Glu, Ser, Ile, Tyr, Phe, and Arg. Previous studies have shown that Lys content is positively correlated with α-amylase inhibition, as the side chains of Lys residues can induce conformational changes in α-amylase via hydrophobic interactions, thereby enhancing inhibitory activity [47]. Similarly, hydrolysates enriched in Ile, Tyr, Phe, and Arg tend to exhibit stronger α-amylase inhibition, as these amino acids can interact with the enzyme’s active site and reduce substrate accessibility [23].

The α-glucosidase inhibitory activities of the four protease hydrolysates without ultrasonic treatment are shown in Figure 8B. Among them, FH exhibited the highest inhibition rate (34.67%), followed by AH (27.53%), BH (25.78%), and TH (22.96%). These results are consistent with the findings of Fadimu et al. [23], who reported that lupin protein hydrolysates prepared with flavorzyme displayed strong α-glucosidase inhibitory activity, suggesting the presence of α-glucosidase inhibitory peptides. In general, low-molecular-weight peptides tend to exhibit superior α-glucosidase inhibition due to easier access to the enzyme’s active site [51]. However, Wang et al. [52] found that among four fractions (C1-1, C-1-2, C-1-3, and C-1-4) of Semen Ziziphi Spinosae peptides purified via Sephadex G-25 chromatography, the fraction with the lowest molecular weight (C1-1) showed significantly lower α-glucosidase inhibitory activity than the other fractions. This observation indicates that molecular weight alone cannot serve as the sole determinant of hydrolysate activity; factors such as amino acid composition and structural features must also be considered. Notably, FH contained higher proportions of Tyr, Lys, and Arg compared to the other groups. Tyr enhances the formation of hydrogen bonds with α-glucosidase [53], while Lys and Arg residues strengthen binding to the enzyme through electrostatic interactions [20].

Ultrasonic pretreatment significantly enhanced the α-amylase and α-glucosidase inhibitory activities of the hydrolysates in all groups. In the alcalase and trypsin groups, α-amylase inhibitory activity increased by 18.76% and 22.75%, respectively, at ultrasonic power of 300 W and 500 W. Similarly, in the flavorzyme and bromelain groups, α-glucosidase inhibitory activity increased by 6.88% and 28.69% at an ultrasonic power of 600 W. The potential mechanisms underlying the improvement in hypoglycemic activity by ultrasonic pretreatment can be summarized as follows: (I) the cavitation effect generated by ultrasonication promotes the formation of low-molecular-weight peptides, thereby enhancing the inhibitory activity of the hydrolysates [23]; (II) ultrasonication induces structural modifications in the hydrolysates, exposing active sites and facilitating interactions with enzyme active centers [47]; and (III) ultrasonic treatment increases the content of amino acids associated with hypoglycemic activity, enhancing enzyme specificity and substrate affinity [31].

This is an exploratory study aimed at evaluating the feasibility of using ultrasound-assisted enzymatic hydrolysis to enhance the bioactivity of pearl mussel hydrolysates. While exploring the potential of synergistic ultrasound and enzymatic hydrolysis processing, this study also provides valuable insights for industrial production. However, it is important to acknowledge the limitations of the current research. To begin with, the complex interaction between ultrasound power and enzyme type requires further optimization for industrial-scale production, taking into account factors such as equipment, raw material costs, and energy consumption. Furthermore, the variables established in this study (type of protease and ultrasound power) resulted in a large number of samples, limiting in-depth characterization analysis (such as the identification of active peptide fragments). Lastly, this study was conducted solely in vitro, without validation of in vivo activity or toxicological evaluation, and thus failed to identify its biological characteristics or potential side effects clearly.

### 2.9. Effects of Different Ultrasonic Power and Proteases on the Functional Properties of Pearl Mussel Hydrolysates

#### 2.9.1. OHC

Oil holding capacity (OHC) is a key indicator for evaluating product flavor and texture, particularly in the meat and confectionery industries [54]. As shown in Figure 4C, the OHC of pearl mussel hydrolysate, without ultrasonic treatment, ranged from 83.08% to 238.41%. Variations in OHC are primarily attributed to differences in apparent density, which influences lipid-binding ability [7]. Notably, the AH group exhibited significantly lower OHC than the other groups. Surface hydrophobicity is generally positively correlated with OHC, as exposed hydrophobic amino acids facilitate interactions between hydrolysates and lipids [10]. However, protease specificity, peptide size, and amino acid composition must also be considered when evaluating OHC in practical applications. De Sousa Araujo et al. [46] reported that superworm (*Zophobas morio*) prepared with alcalase (HA), Protamex (HP), and flavor (HF), exhibited the highest OHC in the HF group, which had a lower DH. In the present study, although the AH group exhibited higher surface hydrophobicity and a richer amino acid composition, its lower molecular weight likely limited OHC [54]. Ultrasonic pretreatment increased the OHC of all groups, with the most significant improvements observed in UBH-6 (226.61%) and UTH-5 (295.07%) (*p* < 0.05). These results are consistent with those reported by Yu et al. [22], who found that ultrasound enhanced the OHC of Acheta domesticus protein hydrolysates. Ultrasonic treatment modifies protein structure, exposing additional hydrophobic amino acids and thereby enhancing lipid-binding capacity [55]. It is noteworthy that OHC does not always increase monotonically with ultrasonic power. For instance, in the alcalase and trypsin groups, OHC initially increased and then decreased as ultrasonic power increased. This trend may result from changes in reaction system conditions, such as temperature and pH, as well as differences in substrate specificity among proteases.

#### 2.9.2. Foaming Capacity and Foaming Stability

Foaming capacity (FC) reflects a sample’s ability to migrate, adsorb, and reorient at the air–water interface [56]. As shown in Figure 4D, the bromelain group exhibited the highest FC (50.51%), whereas the alcalase group showed a significantly lower FC (8.69%) than the other groups (*p* < 0.05). Enzymatic hydrolysis enhances molecular flexibility, promotes protein–protein interactions, and increases the adsorption rate at the air–water interface during foam formation [10]. Differences in FC among the groups likely result from the distinct effects of each protease on substrate molecular structure and surface hydrophobicity. De Souza et al. [7] reported similar findings, noting that porcine liver hydrolysates prepared with alcalase exhibited stronger foaming properties than those prepared with FoodPro PXT. Ultrasonic treatment further increased the FC of hydrolysates, with increases of 44.80% (UFH-6), 59.39% (UAH-3), 68.33% (UBH-6), and 31.69% (UTH-5) in the flavorzyme, alcalase, bromelain, and trypsin groups, respectively. Ultrasound promotes structural modification and exposure of functional groups in hydrolysates while generating small peptides, thereby enhancing their adsorption, migration, and rearrangement at the air–water interface [57].

The results of this study (Figure 4E) indicated that, except for the bromelain group, the foaming stability (FS) of the hydrolysates was generally low. Among these, the FS of the alcalase group was significantly lower than that of the other groups (*p* < 0.05). FS depends on interactions between hydrolysates, and it is generally accepted that stronger interfacial viscoelasticity and thicker interfacial membranes enhance foaming stability. Rong et al. [10] reported that alcalase significantly improved the FS of pumpkin (*Cucurbita moschata*) seed protein more effectively than acidic or neutral proteases, which contrasts with the findings of this study. This discrepancy may be attributed to differences in raw materials and DH. In this study, the DH of the alkaline protease group (37.93%) was significantly higher than that of the pumpkin seed protein hydrolysate (<30%), suggesting the formation of more small peptides. It has been reported that small peptides reduce the thickness and mechanical strength of the air–water interface film by lowering surface tension and solution viscosity [46]. Furthermore, ultrasonic treatment further reduced FS in all hydrolysates. Yang et al. [33] also reported similar findings, noting that ultrasonically generated small peptides negatively affect FS, leading to a decrease in the FS of the housefly (*Musca domestica*) larvae protein hydrolysate.

#### 2.9.3. Emulsion Activity and Emulsion Stability

The emulsion activity index (EAI) is a key indicator of an emulsifier’s adsorption capacity at the oil–water interface. Figure 4F presents the EAI values for the hydrolysates in each group. Without ultrasonic treatment, the EAI values for the four protease hydrolysates ranged from 23.93 m^2^/g to 30.99 m^2^/g. Among them, BH exhibits the highest EAI and AH the lowest. These findings are consistent with Wang et al. [5], who reported that bromelain and trypsin treatments increased the EAI of hempseed protein, whereas alcalase and flavorzyme treatments reduced it. Conversely, another study found that bovine liver hydrolysates prepared with alcalase had significantly higher EAI than those prepared with flavorzyme or trypsin [6]. Such discrepancies may be attributed to variations in raw material sources, protease types, and enzyme-to-substrate ratios. Enzymatic hydrolysis generally regulates the balance between hydrophilic and hydrophobic amino acids and enhances molecular flexibility, thereby facilitating adsorption at the oil–water interface [11]. However, Jin et al. [38] observed that as hydrolysis time increased (15–120 min), the EAI of *Pseudosciaena crocea* roe hydrolysate first increased and then decreased, with a significant reduction at 120 min compared to the control. Although short peptides exhibit excellent migration and adsorption abilities at the oil–water interface, excessive hydrolysis can impair EAI because short peptides are unable to fully unfold and reorganize, preventing the formation of a continuous adsorbed film [7,33]. Ultrasonic treatment effectively enhanced the EAI of pearl mussel hydrolysates. In the flavorzyme and bromelain groups, EAI gradually increased with rising ultrasonic power, peaking at 600 W (33.05 m^2^/g and 43.96 m^2^/g, respectively). These results align with He et al. [56], who reported that ultrasonic treatment improved the EAI of oxhide gelatin peptides. In summary, ultrasonication enhances the migration and adsorption capabilities of peptides at the oil–water interface, and its effect on EAI is closely related to hydrophobicity, molecular flexibility, and particle size.

The resistance of an emulsion to destabilization is a critical determinant of its quality. Figure 4G presents the emulsion stability index (ESI) of the hydrolysates in each group. Significant differences in ESI were observed among hydrolysates prepared with different proteases. Without ultrasonic treatment, the AH group exhibited a significantly lower ESI than the other groups (*p* < 0.05). Moderate hydrolysis is generally considered beneficial for improving ESI. However, excessive hydrolysis reduces ESI, as supported by our results [57]. Short peptides are unable to form a dense, viscoelastic interfacial film at the oil–water interface like longer peptides, and therefore cannot effectively inhibit oil droplet aggregation [7]. Additionally, ultrasonic treatment further decreased the ESI of the pearl mussel hydrolysates. This finding aligns with Zhang et al. [58], who reported that ultrasonic treatment produces low-molecular-weight peptides, which are more likely to form weak interactions at the oil–water interface.

### 2.10. Correlation Analysis

**Figure 9:**
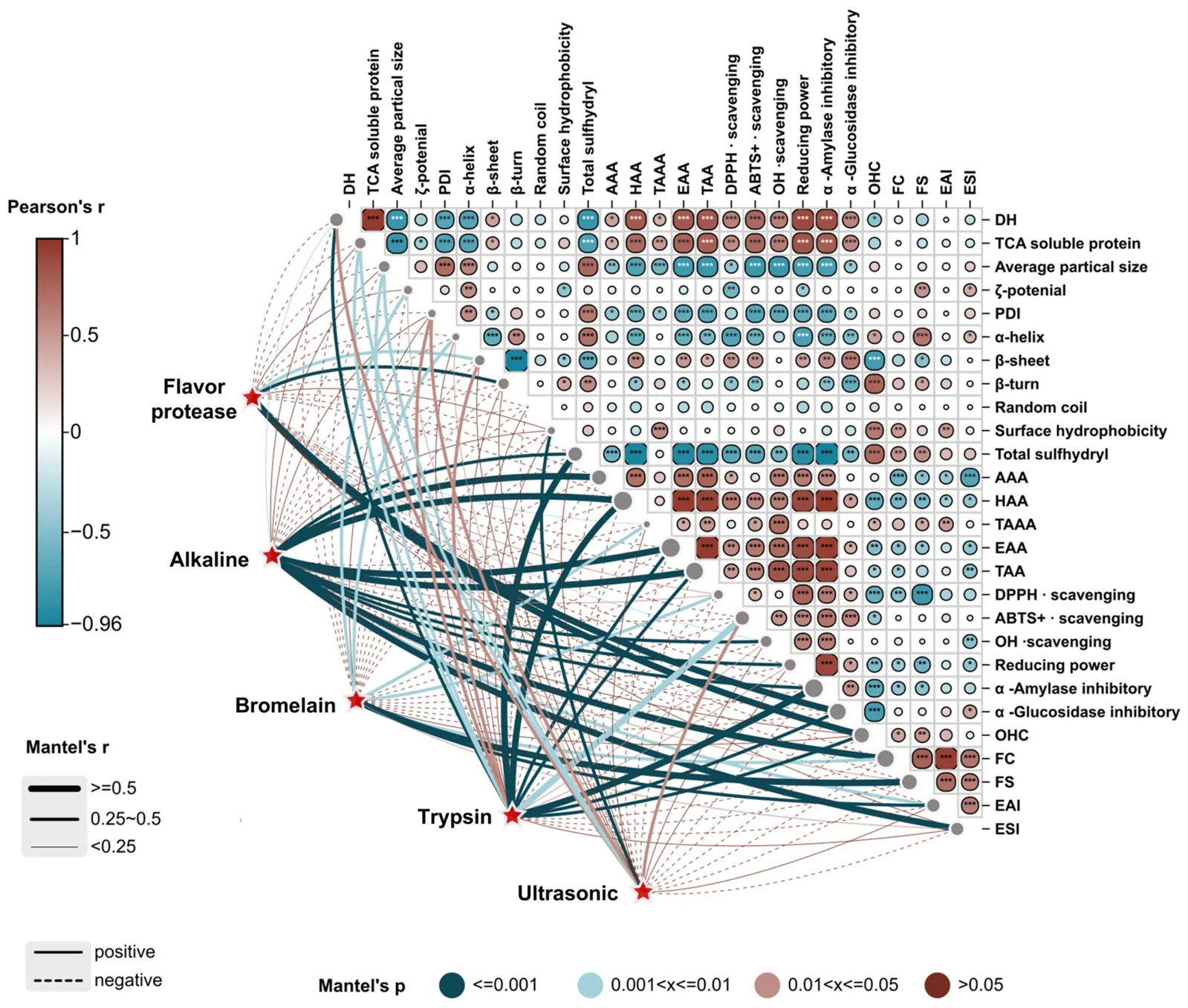
Mantel test for the relationship between different ultrasound powers and protease treatments. In the circles, * indicates *p* < 0.05, ** indicates *p* < 0.01, and *** indicates *p* < 0.001; red indicates a positive correlation, and blue indicates a negative correlation. The thickness of the lines and the size of the circles indicate the magnitude of the difference.

To further investigate the relationship between pearl mussel hydrolysates and processing methods, correlations among hydrolysate indicators, ultrasonic power, and protease type were analyzed using Pearson and Mantel tests. As shown in Figure 9, DH and TCA soluble proteins are significantly positively correlated (*p* < 0.05) with TAAA, EAA, TAA, DPPH radical scavenging activity, ABTS radical scavenging activity, α-amylase inhibitory activity, and α-glucosidase inhibitory activity. This indicates that extensive hydrolysis enhances both the nutritional value and bioactivity of pearl mussel hydrolysates. β-turns and surface hydrophobicity were positively correlated with OHC, FC, and EAI but negatively correlated with ESI, suggesting that hydrolysate functionality is largely determined by structural changes. Furthermore, the correlation analysis indicated that antioxidant and hypoglycemic activities were negatively correlated with OHC, FC, and EAI, implying that while the formation of short peptides maximizes bioactive potential, it may compromise interfacial interactions [5,19]. Mantel test results showed that ultrasonic treatment was positively correlated with DH (*r* = 0.255, *p* = 0.014), TCA soluble protein (*r* = 0.296, *p* = 0.004), β-sheet (*r* = 0.122, *p* = 0.132), and β-turn (*r* = 0.155, *p* = 0.092), surface hydrophobicity (*r* = 0.334, *p* = 0.001), hydroxyl radical scavenging activity (*r* = 0.128, *p* = 0.06), and EAI (*r* = 0.002, *p* = 0.421). Alcalase was positively correlated with AAA (*r* = 0.848, *p* = 0.001), HAA (*r* = 0.630, *p* = 0.001), EAA (*r* = 0.613, *p* = 0.001), TAA (*r* = 0.649, *p* = 0.001), and α-amylase (*r* = 0.453, *p* = 0.001). Bromelain was positively correlated with FC (*r* = 0.392, *p* = 0.003), FS (*r* = 0.881, *p* = 0.001), EAI (*r* = 0.444, *p* = 0.001), and ESI (*r* = 0.153, *p* = 0.03). These findings highlight the interaction between ultrasonic treatment and the hydrolytic capacity, structural characteristics, and biological activity of hydrolysates. Alcalase primarily influences amino acid profiles, while bromelain contributes mainly to hydrolysate functionality.

## 3. Materials and Methods

### 3.1. Materials

The pearl mussels were provided by the College of Fisheries and Life Science at Shanghai Ocean University. Flavorzyme (lot number: 1123J032, enzyme activity: 30 U/mg) was obtained from Beijing Solabio Technology Co., Ltd. (Beijing, China), and alcalase (lot number: M06IS208651, enzyme activity: 200 U/mg) was purchased from Shanghai Yuanye Biotechnology Co., Ltd. Bromelain (lot number: C18352035, enzyme activity: 300 U/mg) and trypsin (lot number: C17529601, enzyme activity: 75 U/mg), o-phthalaldehyde, bromophenol blue, and sodium tetraborate were obtained from Shanghai McLean Biochemical Technology Co., Ltd. (Shanghai, China). KBr, 1,1-diphenyl-2-picrylhydrazyl (DPPH), and 2,2′-azino-bis(3-ethylbenzothiazoline-6-sulfonic acid (ABTS) were obtained from Beijing Solabio Technology Co., Ltd. (Beijing, China). All other reagents were of analytical grade and commercially available.

### 3.2. Preparation of Pearl Mussel Hydrolysate

Fresh pearl mussels were shucked, retaining the meat after removal of the viscera. The meat was freeze-dried and ground into powder using an Antcoa JC-FE-200 crusher (Beijing, China).

The resulting powder was dissolved in 10 volumes of deionized water and subjected to ultrasonic pretreatment. Using an ultrasonic cell disruptor (JY92-IIN, Ningbo, China), samples were treated for 10 min in pulsed mode at a frequency of 20 kHz, with power levels of 100, 200, 300, 400, 500, and 600 W (2 s on, 8 s off). Hydrolysis was subsequently performed using flavorzyme (pH 7.0, 50 °C), alcalase (pH 8.5, 50 °C), bromelain (pH 7.0, 55 °C), and trypsin (pH 8.0, 37.5 °C) under their respective optimal conditions, with an enzyme-to-substrate ratio of 10,000 U/g for each group. After 6 h of hydrolysis, the reaction was terminated by placing the samples in boiling water for 10 min. Following cooling to room temperature, the samples were centrifuged at 8000× *g* for 10 min to collect the supernatant, which was then freeze-dried to obtain the final pearl mussel hydrolysates.

In this study, FH, AH, BH, and TH denote pearl mussel hydrolysates prepared with flavorzyme, alcalase, bromelain, and trypsin, respectively, under non-ultrasonic treatment conditions. Similarly, UFH, UAH, UBH, and UTH represent pearl mussel hydrolysates prepared with the same enzymes following ultrasonic pretreatment.

### 3.3. Degree of Hydrolysis

Following the protocol of Quan et al. [19], the o-phthalaldehyde (OPA) method was employed for analysis. Briefly, 0.2 mL of the sample was mixed with 4.0 mL of OPA reagent (80 mg of OPA dissolved in 2.0 mL of ethanol, followed by the addition of 100 mg of SDS, 88 mg of DTT, and 3.81 g of sodium tetraborate, and diluted to 100 mL with deionized water). The mixture was allowed to react at room temperature for 2 min, and the absorbance was subsequently measured at 340 nm using a Shanghai Flash Spectrum SuPerMax 3100 microplate reader (Shanghai, China). Distilled water and L-serine were used as the control and blank groups, respectively.

### 3.4. TCA Soluble Protein Concentration

TCA-soluble protein concentration was determined according to the method described by Magalhães et al. [27]. Briefly, 0.5 mL aliquots of the hydrolysates were collected at 5, 15, 30, 60, 90, 120, 180, 240, 300, and 360 min and mixed with 0.5 mL of 20% TCA to terminate the reaction. The mixtures were subsequently centrifuged at 7500× *g* for 10 min, and the soluble protein content of the supernatants was determined using the Lowry method.

### 3.5. Structural Characterization of Pearl Mussel Hydrolysate

#### 3.5.1. Particle Size and ζ-Potential

Particle size and ζ-potential were determined according to the method described by Tan et al. [35]. The hydrolysates were prepared at a concentration of 1 mg/mL, and their particle size and ζ-potential were measured using a Zetasizer Pro particle size and ζ-potential analyzer (Malvern Napaco, Worcestershire, UK).

#### 3.5.2. Ultraviolet–Visible Spectra and Intrinsic Fluorescence

The method described by Rong et al. [10] was slightly modified. An L5S UV-Vis spectrophotometer (Shanghai Yidian Analytical Instrument Co., Ltd., Shanghai, China) was used to record the UV spectra of the sample (1.0 mg/mL) in the 200–600 nm range.

An F-4600 Hitachi fluorescence spectrometer (Hitachi High-Technologies Corporation, Tokyo, Japan) was used to measure the intrinsic fluorescence spectra of the sample (1.0 mg/mL) at excitation wavelengths of 280 nm and in the 300–500 nm range [31].

#### 3.5.3. Fourier Transform Infrared (FTIR) Spectroscopy

FTIR analysis was performed according to the method described by Zhang et al. [11]. Freeze-dried samples were mixed with KBr at a ratio of 1:100 (*w*/*w*), ground uniformly, and pressed into thin pellets. FTIR spectra were recorded over the range of 400–4000 cm^−1^ at a resolution of 4 cm^−1^ using a Nicolet iS5 Fourier transform infrared spectrometer (Thermo Scientific, Baden-Württemberg, Germany). The secondary structures of the samples were subsequently analyzed using PeakFit 4.12 software.

#### 3.5.4. Surface Hydrophobicity

Surface hydrophobicity was determined according to the method described by Yan et al. [29]. Briefly, 1.0 mL of the sample was mixed with 0.2 mL of bromophenol blue solution (1.0 mg/mL), and the mixture was allowed to react in the dark for 20 min. The mixture was then centrifuged at 5000× *g* for 10 min, and the supernatant was collected for absorbance measurement at 595 nm. An equal volume of deionized water was used in place of the sample as the blank control. Surface hydrophobicity was calculated using the following equation:

(1) $$Surface\;hydrophobicity\left(ug/mg\right)=200\times \frac{\left(A0-A1\right)}{A0}$$

Note: A0 represents the absorbance of the blank group, and A1 represents the absorbance of the sample group.

#### 3.5.5. Total Sulfhydryl Content

Total sulfhydryl content was determined according to the method described by Wang et al. [5]. Briefly, 1.0 mL of the sample (1.0 mg/mL) was mixed with 9.0 mL of 0.05 M phosphate buffer containing 8 M urea (pH 7.0). Subsequently, 0.4 mL of 10 mM DTNB reagent was added, and the mixture was incubated in the dark for 30 min. The absorbance was then measured at 412 nm. The total sulfhydryl content was calculated using the following equation:

(2) $$Total\;sulfhydryl\;content\left(umol/g\right)=10^{6}\times \frac{A412\times D}{\varepsilon \times C}$$

Note: A412 represents the absorbance of the sample after removing the blank; D indicates the dilution factor; ε represents the molar extinction coefficient (136,000 M^−1^ cm^−1^); C represents the protein concentration (mg/mL).

#### 3.5.6. Scanning Electron Microscope (SEM)

The microstructures of the hydrolysates were examined according to the method described by Wang et al. [5]. Freeze-dried samples were mounted on specimen stubs using conductive adhesive and subsequently sputter-coated with gold. The morphological characteristics of the samples were observed using a Hitachi SU5000 scanning electron microscope (Hitachi, Ltd., Kyoto, Japan).

#### 3.5.7. Amino Acid Composition

Amino acid composition was determined according to the method described by de Sousa Araujo et al. [46]. Briefly, 0.2 g of the sample was mixed thoroughly with 10 mL of 6 mol/L HCl. The mixture was evacuated using a vacuum pump, purged with nitrogen, and hydrolyzed at 105 °C for 22 h. After cooling to room temperature, the hydrolysate was adjusted to a final volume of 50 mL. Subsequently, 5.0 mL of the hydrolysate was vacuum-dried at 50 °C and redissolved, and this procedure was repeated twice. The resulting residue was dissolved in 5.0 mL of sample diluent and filtered through a 0.22 μm membrane. Amino acid contents were determined using a Hitachi LA8080 fully automated amino acid analyzer (Hitachi High-Technologies Corporation, Tokyo, Japan).

### 3.6. Antioxidant Activity

#### 3.6.1. DPPH Radical Scavenging Activity

For the DPPH radical scavenging assay, 0.2 mL of the sample at different concentrations (0.1, 0.5, 1, 2, 4, 6, 8, and 10 mg/mL) was mixed with 20.0 μL of DPPH solution (0.2 mg/mL in 95% ethanol). The mixture was incubated in the dark for 30 min, after which the absorbance was measured at 520 nm. DPPH radical scavenging activity was calculated using the following equation:

(3) $$DPPH\;radical\;scavenging\;activity\left(\%\right)=\frac{\left(A0-A1+A2\right)}{A0}\times 100$$

Note: A0 represents the absorbance of the deionized water and DPPH solution group; A1 represents the absorbance of the sample and DPPH solution group; A2 represents the absorbance of the sample and 95% ethanol group.

#### 3.6.2. ABTS Radical Scavenging Activity

For the ABTS radical scavenging assay, 0.1 mL of the sample at different concentrations (0.1, 0.5, 1, 2, 4, 6, 8, and 10 mg/mL) was mixed with 0.4 mL of ABTS working solution. The mixture was incubated in the dark for 10 min, and the absorbance was subsequently measured at 734 nm. ABTS radical scavenging activity was calculated using the following equation:

(4) $$ABTS\;radical\;scavenging\;activity\left(\%\right)=\frac{\left(A0-A1\right)}{A0}\times 100$$

Note: A0 represents the absorbance of the deionized water and ABTS working solution group; A1 represents the absorbance of the sample and ABTS working solution group.

#### 3.6.3. Hydroxyl Radical Scavenging Activity

Hydroxyl radical scavenging activity was determined according to the method described by Hu et al. [49]. Briefly, 2.0 mL of the sample at different concentrations (1, 5, 10, 20, 30, and 40 mg/mL) was mixed thoroughly with 2.0 mL of 9 mmol/L FeSO_4_, 2.0 mL of 9 mmol/L salicylic acid, and 8.8 mmol/L H_2_O_2_. The mixture was incubated in a water bath at 37 °C for 20 min, after which the absorbance was measured at 510 nm. Hydroxyl radical scavenging activity was calculated using the following equation:

(5) $$Hydroxyl\;radical\;scavenging\;activity\left(\%\right)=1-\frac{\left(A1-A2\right)}{A0}\times 100$$

Note: A0 represents the absorbance of the deionized water substitute in the sample group; A1 represents the absorbance of the sample group; A2 represents the absorbance of the deionized water substitute in the H_2_O_2_ group.

#### 3.6.4. Reducing Power

Reducing power was determined according to the method described by Jin et al. [38]. Briefly, 0.5 mL of the sample at different concentrations (0.1, 0.5, 1, 2, 4, 6, 8, and 10 mg/mL) was mixed with 2.5 mL of 0.2 M phosphate buffer and 2.5 mL of 1% potassium ferricyanide. The mixture was incubated in a water bath at 50 °C for 20 min and subsequently cooled to room temperature. Thereafter, 2.5 mL of 10% trichloroacetic acid was added, and the mixture was centrifuged at 4000× *g* for 10 min. Subsequently, 1.0 mL of the supernatant was mixed with 0.2 mL of 0.1% FeCl_3_ and allowed to stand for 10 min. The absorbance was then measured at 700 nm [38].

### 3.7. Hypoglycemic Activity

#### 3.7.1. α-Amylase Inhibitory Activity

α-Amylase inhibitory activity was determined according to the method described by Du et al. [31]. Briefly, 0.2 mL of the sample at different concentrations (1, 2, 4, 6, and 8 mg/mL), prepared in 0.1 M phosphate buffer (pH 7.2), was mixed with 0.1 mL of α-amylase solution (1 U/mL) and incubated in a water bath at 37 °C for 15 min. Subsequently, 5.0 mL of 1% soluble starch solution was added, and the mixture was incubated at 37 °C for an additional 15 min. Thereafter, 6.0 mL of 3,5-dinitrosalicylic acid (DNS) reagent was added, and the mixture was heated in boiling water for 15 min. After cooling to room temperature, the absorbance was measured at 540 nm. α-Amylase inhibitory activity was calculated using the following equation:

(6) $$\alpha \text{-}amylase\;inhibitory\;activity\left(\%\right)=1-\frac{\left(A1-A2\right)}{\left(A3-A4\right)}\times 100$$

Note: A1 represents the absorbance of the sample group; A2 represents the absorbance of the sample background group; A3 represents the absorbance of the control group; A4 represents the absorbance of the control background group.

#### 3.7.2. α-Glucosidase Inhibitory Activity

α-Glucosidase inhibitory activity was determined according to the method described by Wang et al. [52]. Briefly, 0.2 mL of the sample at different concentrations (1, 2, 4, 6, and 8 mg/mL), prepared in 0.2 M phosphate buffer (pH 6.8), was mixed with 0.1 mL of α-glucosidase solution (0.2 U/mL) and 0.5 mL of phosphate buffer. The mixture was incubated in a water bath at 37 °C for 30 min. Subsequently, 0.4 mL of 5 mM 4-nitrophenyl-β-D-glucopyranoside (p-NPG) solution was added, and the mixture was incubated at 37 °C for another 30 min. The reaction was terminated by adding 1.3 mL of 0.2 M Na_2_CO_3_, and the absorbance was measured at 412 nm. α-Glucosidase inhibitory activity was calculated using the following equation:

(7) $$\alpha \text{-}glucosidase\;inhibitory\;activity\left(\%\right)=1-\frac{\left(A1-A2\right)}{\left(A3-A4\right)}\times 100$$

Note: A1 represents the absorbance of the sample group; A2 represents the absorbance of the sample background group; A3 represents the absorbance of the control group; A4 represents the absorbance of the control background group.

### 3.8. Functional Properties

#### 3.8.1. Oil Holding Capacity (OHC)

Oil holding capacity (OHC) was determined according to the method described by de Souza et al. [7]. Briefly, 1.0 g of the sample was mixed with 5.0 mL of soybean oil in a 10 mL centrifuge tube, vortexed for 1 min, and allowed to stand for 30 min. The mixture was then centrifuged at 2000× *g* for 15 min, after which the supernatant was removed and the tube was weighed. OHC was calculated using the following equation:

(8) $$Oil\;holding\;capacity\left(\%\right)=\frac{\left(A1-A0\right)}{A0}\times 100$$

Note: A0 represents the initial sample weight; A1 represents the final weight after centrifugation, excluding the weight of the supernatant and the empty centrifuge tube.

#### 3.8.2. Foaming Capacity and Foaming Stability

Following the method described by Wu et al. [59], 20 mL of a 20 mg/mL sample solution was homogenized at 18,000 rpm for 1 min, then quickly transferred to a 50 mL graduated cylinder, where the volume was recorded. The sample was then left to stand at room temperature for 30 min, and the volume was recorded again. The foaming capacity (FC) and foaming stability (FS) were calculated using the following equations:

(9) $$FC\left(\%\right)=\frac{\left(A1-A0\right)}{A0}\times 100$$

(10) $$FS\left(\%\right)=\frac{\left(A2-A0\right)}{\left(A1-A0\right)}\times 100$$

Note: A0 represents the sample volume before homogenization; A1 represents the sample volume after homogenization; A2 represents the sample volume after standing for 30 min.

#### 3.8.3. Emulsion Activity and Emulsion Stability

Emulsifying activity were determined according to the method described by Pé-rez-Gálvez et al. [60]. Briefly, 1.0 mL of soybean oil was added to 9.0 mL of the sample solution (20 mg/mL), and the mixture was homogenized at 18,000 rpm for 1 min using an RCD-1A constant-speed homogenizer (Changzhou Hongze Experimental Technology Co., Ltd., Changzhou, China). Rapidly, 40 μL of the emulsion was aspirated from the bottom, add to 4 mL of 1% SDS solution, and mixed thoroughly. The absorbance was measured at 500 nm; then, the mixture was left to stand for 10 min and the absorbance was measured again, using 1% SDS solution as a blank control. The emulsion activity index (EAI) and emulsion stability index (ESI) were calculated using the following equations:

(11) $$EAI\left(m^{2}/g\right)=\frac{\left(2\times 2.303\times N\times A0\right)}{\emptyset \times C\times 10,000}\times 100$$

(12) $$ESI\left(\%\right)=\frac{A1}{A0}\times 100$$

Note: A0 and A1 represent the absorbance at 500 nm of the emulsion at 0 and 10 min; N represents the dilution factor; ∅ represents the volume fraction of the oil phase; and C represents the protein concentration (g/mL).

### 3.9. Statistical Analysis and Graphs

All experiments were repeated three times, and data are presented as mean ± standard deviation. Data analysis was performed using SPSS software (version 26.0, SPSS Inc., Chicago, IL, USA) (one-way ANOVA and Duncan’s multiple range test). Graphs were generated using Origin 2024 (Massachusetts, MA, USA). *p* < 0.05 was considered statistically significant.

## 4. Conclusions

This is a report on pearl mussel hydrolysates. In this study, the effects of ultrasonic power and protease type on the physicochemical, bioactivity, and functional properties of the hydrolysates were systematically evaluated. Ultrasonic treatment significantly increased the DH, reduced particle size and the proportion of α-helices, and enriched the amino acid profile, while simultaneously enhancing antioxidant, hypoglycemic, and emulsion properties. Furthermore, the type of protease used is particularly important. Among the four enzymes tested, alcalase most effectively modulated hydrolysate structure and improved bioactivity, although its hydrolysates exhibited limited capacity to form cohesive interfacial films, indicating the need for further functional optimization. In contrast, bromelain hydrolysates combined excellent hydrophobicity with a favorable amino acid profile, and their moderate DH contributed to superior interfacial properties. Overall, these findings highlight the potential of integrating ultrasonication with enzymatic hydrolysis to improve the functional and bioactive qualities of pearl mussel hydrolysates, providing valuable insights for their application in the food and pharmaceutical industries. Although our findings demonstrate that ultrasonic treatment and protease type play important roles in modulating the characteristics of freshwater mussel protein hydrolysates, the underlying mechanisms remain to be fully elucidated. Future work should focus on process optimization, mechanistic validation, and application expansion to fully exploit their industrial potential.

## Data Availability

The original contributions presented in this study are included in the article. Further inquiries can be directed to the corresponding author.

## Supplementary Materials

The following supporting information can be downloaded at https://www.mdpi.com/article/10.3390/md24100348/s1, Table S1. Amino acid composition (mg/100 g sample) of pearl mussel hydrolysates under different ultrasonic power and protease treatment conditions. Different letters denote the presence of significant differences (*p* < 0.05).
