# Supplementary material for: Valorizing Pearl Mussel (Hyriopsis cumingii) into Hydrolysates: Impact of Different Ultrasonic Pretreatments and Proteases on Its Physicochemical and Functional Properties
Source: Mar Drugs. 2026 Oct 2;24(10):348. doi: 10.3390/md24100348 (PMC13707070; doi:10.3390/md24100348)
Supplement: Supplementary file 1 [file marinedrugs-24-00348-s001.zip › marinedrugs-4484009-supplementary.pdf]

**Table S1.** Amino acid composition (mg/100g sample) of pearl mussel hydrolysates under different ultrasonic power and protease treatment conditions. Different letters denote the presence of significant differences ($p < 0.05$).

| Amino acid | FH | UFH-1 | UFH-2 | UFH-3 | UFH-4 | UFH-5 | UFH-6 |
|---|---|---|---|---|---|---|---|
| Asp | 174.61±1.61$^{c}$ | 177.40±1.04$^{abc}$ | 176.87±1.85$^{bc}$ | 177.30±1.62$^{abc}$ | 178.52±1.80$^{abc}$ | 180.12±0.86$^{ab}$ | 181.35±2.16$^{a}$ |
| Thr | 79.34±0.72$^{b}$ | 80.46±0.67$^{b}$ | 80.18±0.87$^{b}$ | 80.92±0.52$^{ab}$ | 80.53±0.94$^{b}$ | 82.37±0.21$^{a}$ | 82.54±0.81$^{a}$ |
| Ser | 80.54±0.84$^{c}$ | 81.10±0.64$^{bc}$ | 81.91±0.91$^{bc}$ | 82.49±0.92$^{ab}$ | 82.04±0.46$^{bc}$ | 82.40±0.23$^{abc}$ | 83.97±0.68$^{a}$ |
| Glu | 262.76±3.82$^{b}$ | 267.99±3.18$^{ab}$ | 269.34±2.03$^{ab}$ | 268.45±1.25$^{ab}$ | 270.64±4.87$^{ab}$ | 274.13±0.07$^{a}$ | 275.50±2.65$^{a}$ |
| Gly | 103.69±1.53$^{b}$ | 103.92±0.81$^{b}$ | 106.03±1.05$^{ab}$ | 104.28±0.77$^{b}$ | 106.32±1.13$^{ab}$ | 107.29±0.80$^{a}$ | 108.42±1.02$^{a}$ |
| Ala | 104.13±0.50$^{b}$ | 104.59±0.37$^{b}$ | 104.35±0.82$^{b}$ | 104.40±0.44$^{b}$ | 105.10±1.40$^{ab}$ | 105.83±0.58$^{ab}$ | 106.93±0.73$^{a}$ |
| Cys | 4.59±0.23$^{b}$ | 2.60±0.12$^{e}$ | 4.65±0.03$^{b}$ | 4.22±0.05$^{c}$ | 4.30±0.06$^{c}$ | 3.19±0.08$^{d}$ | 5.03±0.15$^{a}$ |
| Val | 88.52±1.08$^{b}$ | 88.31±0.52$^{b}$ | 88.00±0.57$^{b}$ | 88.03±0.40$^{b}$ | 87.89±0.91$^{b}$ | 89.29±0.59$^{ab}$ | 90.53±0.52$^{a}$ |
| Met | 32.02±0.65$^{b}$ | 32.76±0.42$^{b}$ | 33.05±0.40$^{b}$ | 33.29±1.12$^{b}$ | 32.34±0.75$^{b}$ | 32.58±0.31$^{b}$ | 35.11±0.81$^{a}$ |
| Ile | 83.99±1.56$^{b}$ | 85.11±0.85$^{b}$ | 85.01±1.07$^{b}$ | 84.79±0.88$^{b}$ | 83.27±1.84$^{b}$ | 84.77±0.56$^{b}$ | 88.77±0.47$^{a}$ |
| Leu | 143.14±1.95$^{b}$ | 145.94±0.91$^{b}$ | 145.84±2.13$^{b}$ | 145.87±0.25$^{b}$ | 145.81±2.19$^{b}$ | 147.24±1.18$^{ab}$ | 150.51±0.71$^{a}$ |
| Tyr | 61.49±1.34$^{b}$ | 62.57±0.62$^{b}$ | 62.55±1.12$^{b}$ | 62.58±0.68$^{b}$ | 62.52±1.44$^{b}$ | 63.21±0.84$^{b}$ | 65.86±0.14$^{a}$ |
| Phe | 60.74±0.89$^{b}$ | 61.32±0.76$^{b}$ | 61.69±0.91$^{b}$ | 60.98±0.27$^{b}$ | 60.79±0.33$^{b}$ | 61.35±0.82$^{b}$ | 64.28±0.06$^{a}$ |
| Lys | 134.54±1.92$^{ab}$ | 132.79±1.23$^{b}$ | 133.03±1.26$^{b}$ | 133.89±0.67$^{ab}$ | 131.96±3.02$^{b}$ | 135.55±1.37$^{ab}$ | 138.12±1.15$^{a}$ |
| His | 41.28±0.15$^{bc}$ | 42.04±0.61$^{ab}$ | 39.20±0.72$^{d}$ | 40.46±0.91$^{c}$ | 40.89±0.23$^{bc}$ | 42.92±0.37$^{a}$ | 41.01±0.30$^{bc}$ |
| Arg | 143.15±0.93$^{a}$ | 140.46±0.88$^{a}$ | 140.79±1.29$^{a}$ | 142.17±1.22$^{a}$ | 141.75±3.19$^{a}$ | 143.83±0.99$^{a}$ | 144.89±1.76$^{a}$ |
| Pro | 76.77±0.82$^{a}$ | 79.01±0.92$^{ab}$ | 77.67±0.32$^{bc}$ | 76.89±0.52$^{c}$ | 79.23±1.05$^{ab}$ | 78.91±0.10$^{ab}$ | 80.49±0.67$^{a}$ |
| AAA | 122.24±2.23$^{b}$ | 123.89±1.38$^{b}$ | 124.25±2.03$^{b}$ | 123.56±0.95$^{b}$ | 123.30±1.76$^{b}$ | 124.56±1.65$^{b}$ | 130.13±0.19$^{a}$ |
| HAA | 589.31±7.45$^{b}$ | 597.04±4.75$^{b}$ | 595.61±6.22$^{b}$ | 594.25±3.88$^{b}$ | 594.43±8.47$^{b}$ | 599.97±4.14$^{ab}$ | 616.62±3.97$^{b}$ |
| TAAA | 417.07±5.22$^{b}$ | 413.22±3.88$^{b}$ | 413.27±4.82$^{b}$ | 416.61±4.65$^{b}$ | 413.76±8.69$^{b}$ | 421.28±3.96$^{b}$ | 430.02±4.31$^{a}$ |
| EAA | 663.57±8.92$^{b}$ | 668.74±5.98b | 666.00±7.9$^{b}$ | 668.22±5.01$^{b}$ | 663.47±10.20$^{b}$ | 676.07±5.40$^{ab}$ | 690.88±4.81$^{a}$ |
| TAA | 1675.32±20.55$^{b}$ | 1688.37±14.56$^{b}$ | 1690.15±17.36$^{b}$ | 1691.02±12.50$^{b}$ | 1693.88±25.59$^{b}$ | 1714.98±9.94$^{ab}$ | 1743.32±14.76$^{a}$ |

**Table S1.**

| Amino acid | AH | UAH-1 | UAH-2 | UAH-3 | UAH-4 | UAH-5 | UAH-6 |
|---|---|---|---|---|---|---|---|
| Asp | 187.34±1.00$^{b}$ | 187.33±2.16$^{b}$ | 189.43±2.96$^{ab}$ | 193.66±1.51$^{a}$ | 193.18±1.78$^{a}$ | 188.91±0.57$^{ab}$ | 187.91±2.42$^{b}$ |
| Thr | 86.48±1.58$^{a}$ | 86.31±0.80$^{a}$ | 87.13±1.07$^{a}$ | 89.51±1.50$^{a}$ | 88.77±1.28$^{a}$ | 87.09±0.16$^{a}$ | 87.07±1.58$^{a}$ |
| Ser | 89.29±1.17$^{ab}$ | 88.84±0.10$^{b}$ | 88.75±1.96$^{b}$ | 92.48±1.50$^{a}$ | 91.25±1.03$^{ab}$ | 89.27±0.31$^{ab}$ | 89.51±1.40$^{ab}$ |
| Glu | 290.53±6.35$^{b}$ | 292.67±4.06$^{ab}$ | 291.09±5.57$^{ab}$ | 302.59±4.08$^{a}$ | 301.62±2.68$^{ab}$ | 291.74±0.38$^{ab}$ | 292.05±4.27$^{ab}$ |
| Gly | 108.56±1.32$^{abc}$ | 109.45±0.98$^{abc}$ | 111.06±2.61$^{abc}$ | 111.68±1.80$^{ab}$ | 112.44±0.14$^{a}$ | 107.68±0.34$^{c}$ | 108.04±1.42$^{bc}$ |
| Ala | 111.13±0.95$^{a}$ | 111.62±1.92$^{a}$ | 113.12±1.26$^{a}$ | 115.13±2.53$^{a}$ | 114.61±0.93$^{a}$ | 112.79±0.96$^{a}$ | 112.36±2.42$^{a}$ |
| Cys | 6.23±0.10$^{ab}$ | 3.67±0.09$^{d}$ | 5.21±1.52$^{bc}$ | 4.03±0.05$^{cd}$ | 6.85±0.07$^{a}$ | 3.44±0.05$^{d}$ | 5.77±0.49$^{ab}$ |
| Val | 90.97±0.62$^{b}$ | 90.12±0.10$^{b}$ | 94.75±1.17$^{a}$ | 93.09±2.19$^{ab}$ | 91.21±1.15$^{b}$ | 93.19±0.78$^{ab}$ | 91.54±0.90$^{b}$ |
| Met | 34.20±1.19$^{b}$ | 33.11±0.79$^{b}$ | 37.27±1.17$^{a}$ | 33.89±0.55$^{b}$ | 36.06±1.20$^{ab}$ | 33.96±0.27$^{b}$ | 33.97±2.02$^{b}$ |
| Ile | 85.61±1.30$^{b}$ | 84.68±0.13$^{b}$ | 92.48±3.07$^{a}$ | 87.57±1.76$^{ab}$ | 86.85±2.88$^{b}$ | 89.07±0.71$^{ab}$ | 87.12±2.70$^{ab}$ |
| Leu | 153.49±0.01$^{b}$ | 153.71±2.14$^{b}$ | 158.67±2.78$^{a}$ | 159.03±1.32a | 159.12±1.29$^{a}$ | 156.11±1.25$^{a}$ | 155.12±2.83$^{ab}$ |
| Tyr | 61.82±1.09$^{b}$ | 64.67±0.31$^{ab}$ | 68.02±1.99$^{ab}$ | 66.55±1.76$^{ab}$ | 68.59±2.48$^{a}$ | 54.92±0.94$^{c}$ | 63.19±4.93$^{ab}$ |
| Phe | 65.60±0.27$^{ab}$ | 66.39±3.35$^{ab}$ | 68.79±1.33$^{a}$ | 68.58±1.53$^{a}$ | 69.26±2.35$^{a}$ | 63.55±0.97$^{b}$ | 65.82±1.29$^{ab}$ |
| Lys | 141.44±1.10$^{c}$ | 143.17±3.53$^{bc}$ | 147.90±0.14$^{ab}$ | 148.65±3.13$^{a}$ | 148.45±1.03$^{a}$ | 145.01±1.27$^{abc}$ | 142.18±1.64$^{c}$ |
| His | 40.12±0.79$^{b}$ | 43.93±1.93$^{ab}$ | 46.09±3.96$^{a}$ | 44.66±1.27$^{ab}$ | 40.04±1.63$^{b}$ | 46.73±1.35$^{a}$ | 39.87±1.51$^{b}$ |
| Arg | 138.56±1.04$^{b}$ | 140.73±1.82$^{b}$ | 148.37±2.23$^{a}$ | 143.11±2.72$^{ab}$ | 142.06±4.86$^{ab}$ | 143.40±1.29$^{ab}$ | 140.43±1.96$^{b}$ |
| Pro | 82.36±0.89$^{b}$ | 83.65±0.57$^{ab}$ | 83.56±1.13$^{ab}$ | 85.72±1.22$^{a}$ | 85.20±1.65$^{ab}$ | 84.00±1.10$^{ab}$ | 82.565±1.22b |
| AAA | 127.42±1.36$^{a}$ | 131.06±3.66$^{a}$ | 136.81±3.32$^{a}$ | 135.13±3.29$^{a}$ | 137.84±4.83$^{a}$ | 118.47±1.92$^{b}$ | 129.02±6.22$^{a}$ |
| HAA | 623.36±5.23$^{c}$ | 623.28±9.00$^{bc}$ | 648.64±11.91$^{a}$ | 643.01±11.10$^{ab}$ | 642.31±11.45$^{ab}$ | 632.67±6.04$^{bc}$ | 628.49±13.38$^{bc}$ |
| TAAA | 422.37±5.31$^{b}$ | 429.28±8.47$^{b}$ | 452.86±11.01$^{a}$ | 440.89±9.48$^{ab}$ | 442.05±11.27$^{ab}$ | 427.46±5.17$^{ab}$ | 425.41±12.55$^{b}$ |
| EAA | 697.91±6.86$^{b}$ | 701.41±12.77$^{ab}$ | 733.10±14.70$^{a}$ | 724.98±13.25$^{ab}$ | 719.75±12.80$^{ab}$ | 714.70±6.76$^{ab}$ | 720.70±17.47$^{ab}$ |
| TAA | 1773.74±20.78$^{b}$ | 1784.04±14.78$^{b}$ | 1831.71±15.93$^{a}$ | 1839.92±20.42$^{a}$ | 1835.55±18.42$^{a}$ | 1790.84±12.69$^{b}$ | 1784.50±27.99$^{b}$ |

**Table S1.**

| Amino acid | BH | UBH-1 | UBH-2 | UBH-3 | UBH-4 | UBH-5 | UBH-6 |
|---|---|---|---|---|---|---|---|
| Asp | 172.55±5.61[a] | 175.00±1.92[a] | 173.39±11.66[a] | 175.24±2.06[a] | 175.85±7.64[a] | 176.77±4.64[a] | 179.88±5.59[a] |
| Thr | 80.45±4.11[a] | 80.71±0.73[a] | 81.24±6.22[a] | 82.13±1.43[a] | 82.02±3.33[a] | 83.08±6.93[a] | 83.44±2.42[a] |
| Ser | 83.19±9.00[a] | 84.05±0.52[a] | 83.95±4.10[a] | 84.64±0.46[a] | 84.23±2.68[a] | 86.58±3.00[a] | 86.05±3.68[a] |
| Glu | 281.85±26.69[a] | 282.86±3.10[a] | 281.95±17.84[a] | 284.09±6.14[a] | 287.62±13.54[a] | 286.47±21.18[a] | 292.52±8.39[a] |
| Gly | 101.82±4.91[a] | 98.44±0.33[a] | 102.58±2.20[ab] | 107.22±0.14[a] | 105.23±1.55[ab] | 104.19±2.20[ab] | 106.68±4.77[a] |
| Ala | 97.90±6.69[a] | 98.42±1.07[a] | 99.26±10.66[a] | 101.30±0.06[a] | 100.53±3.02[a] | 101.41±3.67[a] | 102.88±4.35[a] |
| Cys | 8.18±0.54[a] | 7.59±0.22[a] | 7.62±1.02[a] | 7.84±0.17[a] | 7.86±0.69[a] | 7.25±0.83[a] | 5.03±0.28[b] |
| Val | 81.82±2.77[a] | 80.31±1.94[a] | 83.25±5.00[a] | 83.12±1.05[a] | 83.25±1.55[a] | 82.86±6.60[a] | 83.88±3.23[a] |
| Met | 35.36±1.52[ab] | 35.34±0.51[ab] | 35.03±1.49[ab] | 36.21±1.00[a] | 36.32±1.82[a] | 36.39±0.42[a] | 32.91±0.82[b] |
| Ile | 77.41±3.68[a] | 77.36±1.67[a] | 78.76±3.17[a] | 77.66±1.13[a] | 78.28±3.70[a] | 78.01±2.67[a] | 78.89±2.88[a] |
| Leu | 140.52±15.26[a] | 143.13±3.14[a] | 141.62±8.05[a] | 140.83±1.09[a] | 142.78±6.71[a] | 143.71±15.23[a] | 146.73±4.24[a] |
| Tyr | 67.86±3.78[a] | 69.97±2.17[a] | 70.25±2.86[a] | 68.16±1.51[a] | 69.49±1.93[a] | 69.95±2.46[a] | 70.66±2.05[a] |
| Phe | 61.91±4.23[a] | 62.19±1.88[a] | 62.90±3.02[a] | 61.34±1.06[a] | 62.76±3.42[a] | 63.27±4.22[a] | 63.80±2.03[a] |
| Lys | 144.27±15.53[a] | 144.30±2.79[a] | 145.76±9.41[a] | 143.47±2.33[a] | 145.44±7.25[a] | 147.37±10.92[a] | 149.05±4.94[a] |
| His | 36.41±2.07[b] | 37.76±0.53[b] | 38.48±1.68[b] | 37.15±0.54[b] | 38.13±1.85[b] | 38.98±2.58[ab] | 42.49±1.56[a] |
| Arg | 144.96±6.04[a] | 148.30±3.17[a] | 147.42±10.02[a] | 145.75±2.78[a] | 148.26±8.03[a] | 148.62±7.46[a] | 150.87±4.74[a] |
| Pro | 78.36±5.52[a] | 75.24±2.49[a] | 78.94±3.81[a] | 78.74±2.96[a] | 78.71±2.31[a] | 81.27±5.33[a] | 82.97±3.64[a] |
| AAA | 129.77±8.00[a] | 132.16±4.05[a] | 133.15±5.88[a] | 129.50±2.57[a] | 132.25±5.35[a] | 133.22±6.67[a] | 134.46±4.08[a] |
| HAA | 573.28±39.67[a] | 571.99±12.70[a] | 579.76±35.20[a] | 579.20±8.35[a] | 582.63±22.53[a] | 586.92±38.17[a] | 592.06±21.19[a] |
| TAAA | 437.04±9.48[a] | 443.26±9.39[a] | 444.56±16.48[a] | 438.58±8.33[a] | 445.50±21.57[a] | 448.56±7.67[a] | 451.01±14.35[a] |
| EAA | 658.13±50.17[a] | 661.09±13.20[a] | 667.02±41.04[a] | 661.90±9.63[a] | 668.98±29.64[a] | 673.68±51.57[a] | 681.48±24.13[a] |
| TAA | 1694.81±12.94[b] | 1700.97±28.20[ab] | 1712.40±21.21[ab] | 1714.87±25.91[ab] | 1726.75±31.02[ab] | 1736.19±20.35[ab] | 1759.01±21.62[a] |

**Table S1.**

| Amino acid | TH | UTH-1 | UTH-2 | UTH-3 | UTH-4 | UTH-5 | UTH-6 |
|---|---|---|---|---|---|---|---|
| Asp | 161.84±12.24$^{a}$ | 164.29±18.63$^{a}$ | 162.22±17.99$^{a}$ | 170.54±13.97$^{a}$ | 168.84±8.86$^{a}$ | 170.93±3.64$^{a}$ | 164.06±6.97$^{a}$ |
| Thr | 77.07±5.17$^{a}$ | 76.91±4.66$^{a}$ | 78.34±4.19$^{a}$ | 77.87±5.33$^{a}$ | 79.99±4.44$^{a}$ | 81.70±1.50$^{a}$ | 77.87±3.00$^{a}$ |
| Ser | 77.93±5.33$^{a}$ | 79.43±4.30$^{a}$ | 78.21±5.42$^{a}$ | 79.71±6.70$^{a}$ | 80.76±4.32$^{a}$ | 80.53±0.81$^{a}$ | 76.78±3.57$^{a}$ |
| Glu | 267.49±6.49$^{b}$ | 268.83±5.33$^{b}$ | 268.21±2.23$^{b}$ | 278.23±4.39$^{ab}$ | 275.90±6.26$^{ab}$ | 283.60±6.64$^{a}$ | 271.58±5.58$^{ab}$ |
| Gly | 102.46±5.82$^{a}$ | 102.12±8.18$^{a}$ | 104.02±6.03$^{a}$ | 100.78±7.72$^{a}$ | 97.88±3.50$^{a}$ | 103.25±0.55$^{a}$ | 100.61±4.86$^{a}$ |
| Ala | 93.53±0.69$^{a}$ | 93.63±1.07$^{a}$ | 94.74±1.55$^{a}$ | 94.91±0.06$^{a}$ | 95.19±1.02$^{a}$ | 97.05±1.67$^{a}$ | 93.38±1.35$^{a}$ |
| Cys | 7.27±0.39$^{ab}$ | 6.15±0.50$^{c}$ | 7.18±0.71$^{ab}$ | 7.37±0.99$^{ab}$ | 5.01±0.14$^{bc}$ | 7.94±0.06$^{a}$ | 6.48±0.11$^{b}$ |
| Val | 74.84±4.27$^{ab}$ | 75.04±1.84$^{ab}$ | 74.07±3.21$^{ab}$ | 71.73±1.94$^{b}$ | 76.30±3.10$^{ab}$ | 79.56±1.05$^{a}$ | 76.89±1.09$^{ab}$ |
| Met | 34.07±2.93$^{a}$ | 32.88±3.80$^{a}$ | 33.79±4.12$^{a}$ | 33.69±1.35$^{a}$ | 33.66±1.16$^{a}$ | 33.35±1.30$^{a}$ | 34.31±0.89$^{a}$ |
| Ile | 71.42±1.63$^{abc}$ | 71.05±2.12$^{bc}$ | 69.99±0.70$^{bc}$ | 66.93±3.48$^{c}$ | 74.62±1.03$^{ab}$ | 76.58±1.54$^{a}$ | 73.29±2.59$^{ab}$ |
| Leu | 131.95±1.23$^{b}$ | 132.00±1.46$^{b}$ | 131.90±3.44$^{b}$ | 135.07±4.96$^{ab}$ | 139.35±0.92$^{ab}$ | 141.44±3.76$^{a}$ | 134.66±1.54$^{ab}$ |
| Tyr | 65.23±5.34$^{a}$ | 64.95±3.38$^{a}$ | 66.58±7.26$^{a}$ | 64.84±6.68$^{a}$ | 69.37±3.58$^{a}$ | 70.10±2.02$^{a}$ | 67.91±2.49$^{a}$ |
| Phe | 60.05±1.25$^{ab}$ | 60.53±0.90$^{ab}$ | 60.00±1.75$^{ab}$ | 58.44±2.07$^{b}$ | 62.73±1.36$^{ab}$ | 64.08±1.56$^{a}$ | 61.46±1.93$^{ab}$ |
| Lys | 131.60±4.88$^{a}$ | 133.56±1.78$^{ab}$ | 137.00±3.95$^{ab}$ | 135.57±5.28$^{ab}$ | 142.67±4.87$^{ab}$ | 143.24±4.07$^{a}$ | 139.84±3.18$^{ab}$ |
| His | 37.02±3.80$^{ab}$ | 38.53±1.08ab | 37.51±4.87$^{ab}$ | 35.56±2.42$^{b}$ | 44.02±1.76$^{a}$ | 40.14±0.54$^{ab}$ | 39.88±1.69$^{ab}$ |
| Arg | 134.92±10.13$^{b}$ | 137.19±8.72$^{ab}$ | 137.80±11.88$^{b}$ | 145.52±11.42$^{ab}$ | 145.39±8.83$^{ab}$ | 147.35±4.92$^{a}$ | 142.73±5.45$^{ab}$ |
| Pro | 78.81±5.30$^{ab}$ | 78.13±3.76$^{ab}$ | 79.58±2.67$^{b}$ | 76.55±3.35$^{ab}$ | 77.08±3.18$^{ab}$ | 70.25±0.76$^{b}$ | 77.67±1.94ab |
| AAA | 125.28±10.59$^{a}$ | 125.47±12.28$^{a}$ | 126.58±14.02$^{a}$ | 123.28±10.76$^{a}$ | 132.10±6.94$^{a}$ | 134.19±3.59$^{a}$ | 129.37±4.43$^{a}$ |
| HAA | 544.67±5.30$^{b}$ | 543.26±14.95$^{ab}$ | 544.07±17.44$^{ab}$ | 537.32±17.21$^{ab}$ | 558.93±11.77$^{ab}$ | 562.31±3.64$^{a}$ | 511.66±7.33$^{ab}$ |
| TAAA | 410.11±27.47$^{ab}$ | 413.26±19.26$^{ab}$ | 419.86±3.79$^{ab}$ | 422.55±8.13$^{b}$ | 440.12±20.35$^{ab}$ | 442.12±12.91$^{a}$ | 431.15±5.81$^{ab}$ |
| EAA | 618.01±9.17$^{b}$ | 620.48±8.64$^{b}$ | 622.61±14.23$^{b}$ | 614.84±12.82$^{b}$ | 653.35±6.63$^{a}$ | 660.09±5.31$^{a}$ | 638.19±14.92$^{b}$ |
| TAA | 1607.50±23.20$^{b}$ | 1615.20±10.85$^{b}$ | 1621.14±12.67$^{b}$ | 1633.29±6.91$^{b}$ | 1668.77±10.13$^{a}$ | 1691.10±5.17$^{a}$ | 1639.34±4.33$^{b}$ |

Note: Asp: aspartic acid; Thr: threonine; Ser: serine; Glu: glutamic acid; Gly: glycine; Ala: alanine; Cys: cysteine; Val: valine; Met: methionine; Ile: isoleucine; Leu: leucine; Tyr: tyrosine; Phe: phenylalanine; Lys: lysine; His: histidine; Arg: arginine; Pro: proline. AAA: Sum of the quantified aromatic amino acids (Tyr and Phe); HAA: Sum of the quantified hydrophobic amino acids (Ala, Pro, Val, Ile, Met, Leu, and Phe); TAAA: Sum of the quantified antioxidant associated amino acids (Cys, Met, Tyr, His, Lys, and Arg); EAA: Sum of the quantified essential amino acids (Thr, Val, Met, Ile, Leu, Phe, His, and Lys); TAA: Total quantified amino acids.
